# MXenes as Heterogeneous Thermal Catalysts: Regioselective Anti-Markovnikov Hydroamination of Terminal Alkynes with 10^2^ h^–1^ Turnover Frequencies

**DOI:** 10.1021/jacs.4c13481

**Published:** 2025-01-21

**Authors:** Rubén Ramírez Grau, Pablo Garcia-Aznar, German Sastre, Sara Goberna-Ferrón, Octavian Pavel, Alina Tirsoaga, Bogdan Cojocaru, Dana Georgeta Popescu, Vasile I. Parvulescu, Ana Primo, Hermenegildo García

**Affiliations:** † 16774 Instituto Universitario de Tecnología Química, Universitat Politècnica de València-Consejo Superior de Investigaciones Científicas, Universitat Politècnica de València, Av. De los Naranjos s/n, 46022 Valencia, Spain; ‡ Department of Organic Chemistry, Biochemistry and Catalysis, 61783 University of Bucharest, B-dul Regina Elisabeta 4-12, Bucharest 030016, Romania; § 198818 National Institute of Materials Physics, 405A Atomistilor Street, Magurele 077125, Ilfov, Romania

## Abstract

Due to their conductive properties and optoelectronic tunability, MXenes have revolutionized the area of electrocatalysis and active materials in supercapacitors. In comparison, there are only a few reports on MXenes as thermal catalysts for general organic reactions. Herein, the unprecedented catalytic activity of Ti_3_C_2_ MXene for the hydroamination of alkynes is reported, overcoming the limitations of poor activity, lack of selectivity, and stability, which are generally encountered in the solid catalysts known so far. In the case of Ti_3_C_2_, hydroamination exhibits almost complete selectivity for the anti-Markovnikov *regio*isomer, for both aliphatic amines and less-reactive aromatic amines. Ti_3_C_2_ also efficiently catalyzes intramolecular hydroamination, leading to the formation of indol heterocycles. The catalytic hydroamination of C–C multiple bonds is a reaction with complete atom efficiency that may form C–N bonds from convenient reagents. The maximum number of hydroamination sites on the Ti_3_C_2_ nanosheets is quantified by thermoprogrammed NH_3_ desorption. The measured TOF values are on the order of 10^2^ h^–1^, with the highest TOF value being 350 h^–1^ for 1-hexyne hydroamination by *n* **-**butylamine. Therefore, Ti_3_C_2_ is among the few heterogeneous hydroamination catalysts studied, with its activity per site being comparable to the best hydroamination catalysts reported so far. Density functional theory calculations on the models indicate the cooperation of neighboring Ti atoms in the mechanism. Considering the compositional and structural versatility of MXenes, the present findings open the door for further application of MXenes in other general organic reactions.

## Graphical abstract

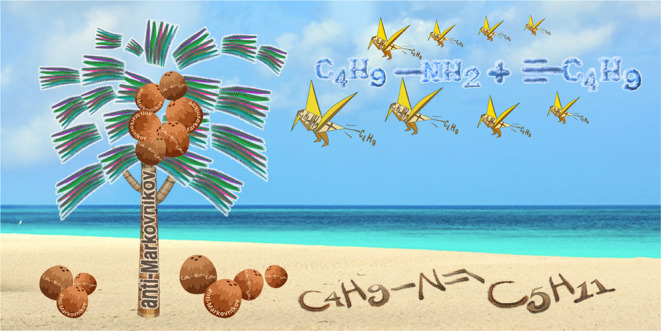

## Introduction

In less than a decade, MXenes have become among the most efficient electrocatalysts for the oxygen reduction (ORR) and evolution (OER) reactions as well as for the hydrogen evolution reaction (HER) and CO_2_ reduction (CO2R), among other electrochemical reactions.^1–3^ The high electrical conductivity combined with a high metal percentage are two favorable properties that have contributed to the growth of this area, consolidating MXenes as efficient electrocatalysts.^4–6^ Besides electrocatalysis, MXenes are also increasingly used in photocatalysis.^7–9^ While conductive MXenes can replace noble metal cocatalysts in photocatalytic systems using dyes or semiconductors as photoresponsive components,^10–12^ there is increasing interest in obtaining semiconducting MXenes with appropriate band alignment to exploit their intrinsic photocatalytic activity.^13,14^ Besides electro- and photocatalysis, MXenes are increasingly used as components in supercapacitors and batteries due to their excellent performance.^15–18^

MXenes are two-dimensional (2D) nanomaterials composed of a few sheets of a one-atom-thick layer of early transition metals alternating with a one-atom-thick layer of carbide, nitride, or carbonitride.^19,20^ The metal layers are always external, and they have surface terminal groups. The general formula of MXenes is M_*n*+1_X*_n_*, where M represents the early transition metal, X is C, N, or C and N in various proportions, and *n* is typically equal to or lower than 3. The bonds between the layers have covalent and ionic characteristics, resulting in unique physical properties and chemical versatility.

The external metal layers are bonded to surface terminal groups, whose nature depends on the preparation procedure. The properties of MXenes are largely dictated by the surface terminal groups. It is well established that the surface terminal groups modulate the physical and electrocatalytic properties of MXenes.^21,22^ For the MXenes prepared by Al etching with F-containing acid solutions of the corresponding MAX phase, the surface groups bonded to the external metal layers are a combination of F, O, and OH terminations.^15^ The concept of the present work is that the surface terminal groups bonded to the metal, their vacancies, defects, and peripheral atoms can become active sites in thermal catalysis able to interact with adsorbates, thereby promoting chemical reactions.^21^

The burst on the exploration of the potential that MXenes offer in many areas sharply contrasts with the so far poor interest that MXenes have received as thermal catalysts.^23,24^ Following the lead in electrocatalysis, there are several reports in which MXenes have been used as 2D supports for metal nanoparticles and even single metal atoms for hydrogenation and dehydrogenation reactions.^25–28^ However, exploitation of the intrinsic structural active sites that MXenes may have in heterogeneous catalysis to promote chemical reactions in the absence of additional metals remains almost unexplored.^29–33^ This is somewhat surprising considering the versatility of MXenes regarding composition, structure, tunability of the surface terminal groups, and the general activity of transition metal compounds as thermal catalysts.

In contrast with the few scattered experimental studies, several theoretical calculations have predicted that MXenes could be efficient thermal catalysts for some particular reactions. In this regard, density functional theory (DFT) calculations suggest that surface-free MXenes adsorb high amounts of H_2_ ^34^ and CO_2,_ ^35^ making them suitable for CO_2_ hydrogenation. Thus, first-principles calculations predict that hydrogen affinity, as an intrinsic property of –O– and −OH– terminated Ti_2_C MXenes, should correlate with the activity of these 2D nanomaterials for C–H activation in light alkane dehydrogenation.^36^ Similarly, DFT-based calculations indicate that the most stable (100) surface of nonstoichiometric Ti_2_C may become a plausible catalyst for methane activation in combination with C-vacancies acting as reaction centers.^37^ However, most of these theoretical studies consider surface-free MXenes, which in most cases are not realistic models due to the universal presence of surface terminal groups.

Given the current state of the art, it is clear that considerably more effort should be devoted to exploring the uncharted opportunities that MXenes offers in thermal catalysis. Besides DFT predictions, chemical intuition may consider MXenes as potential catalysts for those chemical reactions already reported for bulk transition metal carbides,^38^ metallocenes,^39^ and other related early transition complexes having some common structural traits with MXenes.^40^

The present study aims to demonstrate the remarkable catalytic activity of Ti_3_C_2_ MXene to promote the hydroamination of alkynes,^41^ an important reaction in organic synthesis for the formation of imines, enamines, and secondary/tertiary amines. Hydroamination serves to obtain key intermediates in the preparation of antibiotics (β-lactams), antihistaminic drugs (benadryl and chlortrimeton), anticancer agents (carboplatin, camptothecin, and vinblastine), alkaloids as well as some nitrogen heterocycles, among other N-containing organic compounds with biological activity.^42^

Catalytic hydroamination results in the formation of C–N bonds with complete atom efficiency without byproduct generation, thus meeting the strictest requirements of green chemistry.^43^ In comparison with other electrophilic additions to C–C multiple bonds that are catalyzed by acids, nitrogen basicity makes the use of Brönsted or Lewis acids problematic as catalysts since they react preferentially with the amine, leading to their deactivation. Other catalyst types, such as supported noble metal nanoparticles^44–46^ and metal complexes,^47^ have been alternatively used as hydroamination catalysts.

Most of the hydroamination catalysts reported so far are soluble metal complexes, with the heterogeneous catalysts being limited to only a few scattered examples, exhibiting much less activity than soluble complexes.^41,48,49^ However, heterogeneous catalysts offer several advantages over homogeneous catalysts, including facile recovery from the reaction mixture, easy reaction workup, and the possibility of catalyst reuse, which makes them of wide interest.

Ti complexes, including titanocenes having certain structural similarities with Ti_3_C_2_, have been reported among the general homogeneous catalysts for hydroamination.^47,50^ The hypothesis of the present study is that Ti MXenes could also exhibit this type of catalytic activity.^7^ As will be described below, due to their mild acidity and ability to interact with amines, we found that Ti_3_C_2_ MXenes are among the most active and stable heterogeneous solid catalysts reported so far for hydroamination, forming products with almost complete *regio*selectivity. Therefore, the present results uncover the vast potential of MXenes as heterogeneous catalysts for general organic reactions.

## Experimental Section

### Synthesis of Ti_3_C_2_ MXenes

One gram of Ti_3_AlC_2_ (supplied by Chemazone) was added to a solution of 5.96 g of NH_4_F and 40 mL of HCl to etch the Al layers. This mixture was stirred at 50 °C for 24 h, and then the suspension was filtered and washed with Milli-Q water until a pH of 7. The resulting Ti_3_C_2_ was delaminated by ultrasonication (700 W) for 5 h using DMSO as the dispersing agent. Afterward, the material was washed with ethanol to remove as much DMSO as possible and then resuspended in Milli-Q water. Large MXene flakes were decanted, and the supernatant was kept in order to collect the exfoliated layers. Ti_3_C_2_ was obtained from the suspension by drying it at 70 °C overnight in a vacuum system. Chemical analysis of Ti_3_C_2_ MXene indicated Ti and Al contents of 58.02 and 0.70%, respectively.

Ti_2_C was prepared from commercial Ti_2_AlC as reported previously.^51^ Briefly, 2 g of Ti_2_AlC was added to a 12 M HCl solution mixed with 1.3 g of LiF to etch Al. The suspension was magnetically stirred at 50 °C for 24 h. After this time, the resulting solid was filtered and rinsed with water until a pH of 7. Subsequent delamination of the Ti_2_C clay was carried out by stirring the solid in 10 mL of DMSO for 2 h, followed by washing the material with ethanol and recovering the solid by centrifugation. Finally, the Ti_2_C MXene was kept in an aqueous suspension under an Ar atmosphere to minimize spontaneous oxidation.

### Characterization

Textural characteristics (surface area and pore size distribution) were determined from N_2_ adsorption–desorption isotherms at −196 °C using a Micromeritics ASAP 2010 Surface Area and Porosity Analyzer. H_2_ pulsed chemisorption and H_2_ thermoprogrammed desorption (H_2_-TPD) measurements were carried out at a Micromeritics AutoChem II 2920 station. Before H_2_ adsorption, fresh samples were heated at 450 °C at a rate of 10 °C min^–1^ in 30 mL × min^–1^ He flow to desorb moisture and clean the solid surface. Subsequently, the samples were cooled down to room temperature, while maintaining a constant He flow. Afterward, the activated materials were exposed to pulses of 5 vol % H_2_ in He until the peak area corresponding to H_2_ gas became constant and no further H_2_ uptake was observed. Subsequently, H_2_ thermal desorption was carried out by heating the sample at a constant rate of 10 °C min^–1^ in He flow up to 450 °C. The acid–base properties of the investigated catalysts were measured by CO_2_ and NH_3_-TPD using the same Micromeritics instrument. The samples, placed in a U-shaped quartz reactor with a 0.5 cm inner diameter, were pretreated under He (Purity 5.0, Linde) at 120 °C for 1 h and then exposed to a flow of CO_2_ or NH_3_ (SIAD) for 1 h. After that, the samples were purged at room temperature for 20 min with He flow (50 mL min^–1^) in order to remove the physisorbed species. TPD was then started at a heating rate of 5 °C min^–1^ up to 450 °C. The desorbed gas was quantified with a TC detector. The desorbed NH_3_ or CO_2_, expressed as millimoles per gram of catalyst, was determined using a calibration curve. The Supporting Information provides the corresponding raw data for the surface area, pore size distribution, and chemisorption/desorption measurements.

Powder X-ray diffraction (XRD) patterns were recorded on a Shimadzu XRD-7000 diffractometer using Cu Kα radiation (λ = 1.5418 Å, 40 kV, 40 mA) at a scanning rate of 2° × min^–1^ in the 5–90° 2θ angle.

Raman spectra were acquired in the spectral region from 150 nm to 4000 cm^–1^. Raman analysis was carried out with a Horiba Jobin Yvon-Labram HR UV–visible Raman microscope using the excitation wavelengths of 325, 488, 633, and 785 nm.

High-resolution X-ray photoelectron spectroscopy (HR XPS) measurements were performed with an AXIS Ultra DLD (Kratos Surface Analysis) instrument operating under ultrahigh vacuum (10^–9^ mbar basal pressure), equipped with a 165 mm hemispherical analyzer, dual anode (Mg/Al Kα) X-ray source, and monochromatized (Al Kα) X-ray source (*h*ν = 1486.74 eV). In this study, monochromatic (Al Kα) X-rays produced by an X-ray gun operating at 144 W (12 kV × 12 mA) were used. A flood gun (charge balance 2.7 V, filament current 1.5 A, and filament bias 1 V) was employed in order to avoid sample charging effects. The acquisition setup was operated in the spectrum FOV2 mode using 40 eV pass energy with an analyzer aperture of 110 μm. Transmission electron microscopy (TEM) images were acquired in a JEOL JEM 2100F instrument at an accelerating voltage of 200 kV. The specimens were prepared by depositing one microdrop of an ethanolic suspension of the material onto a carbon-coated nickel TEM grid and allowing it to dry at room temperature. Atomic force microscopy (AFM) images were obtained using a Bruker Multimode AFM instrument by depositing a drop of freshly dispersed Ti_3_C_2_ sample in H_2_O on an atomically flat mica surface using the tap method.

### Catalytic Tests

All the starting substrates, including hexynes (1-hexyne, 1-phenyl-1-hexyne, 1-phenylacetylene, and 4-octyne) and amines (aniline, 4-ethylaniline, 2-/3-methylaniline, 3,5-dimethylaniline, 2,6-dimethylaniline, anisidine, 4-aminophenol, *n*-butylamine, and 4-amino-1-butanol) and 2-(phenylethynyl)­(aza)­phenylamines, were purchased from Sigma-Aldrich and used without any further purification.

Hydroamination was carried out by suspending 1 mmol of amine, 2 mmol of 1-hexyne, and 5 mg of Ti_3_C_2_ catalyst in 2 mL of toluene. In the case of the experiment using Ti_2_C, an appropriate volume corresponding to 5 mg of the aqueous Ti_2_C suspension resulting directly from Al leaching was concentrated at low temperature under reduced pressure, and the black solid was recovered with 2 mL of toluene containing 1 mmol of 1-butanamine and 2 mmol of 1-hexyne that were introduced into the reactor. The suspensions were placed in a 7 mL stainless steel reactor. The reaction mixture was heated at 95–160 °C under autogenous pressure and continuous magnetic stirring (800 rpm) for 24 or 48 h, as required. The experiment under pressurized conditions was carried out by first charging and sealing the reactor at room temperature, which was subsequently pressurized with N_2_ at 10 bar before heating. After this time, the autoclave was cooled down at room temperature, the catalyst was recovered by centrifugation, and the reaction products were analyzed using the GC-MS apparatus (Thermo Scientific: Trace 1310 chromatograph coupled with an ISQ LT MS) equipped with a nonpolar GC separation column (TG-5SilMS, 30 m × 0.25 mm × 0.25 μm) with He as the carrier gas. The temperature program was set to 45 °C for a 6 min dwell, then a 10 °C min^–1^ heating ramp up to 240 °C, followed by a 10 min dwell. Mass spectra were recorded in a positive polarization mode, in the range of *m*/*z* 50–300 amu. The turnover frequency (TOF) values were calculated from the number of converted alkyne molecules divided by the number of active sites present on the Ti_3_C_2_ catalyst, calculated from the mmol g_Ti_3_C_2__^–1^ of NH_3_ chemisorption and time, according to the following formula

$$\mathrm{TOF}=\frac{\mathrm{Hydroa}\min\mathrm{ation}\,\;\;\;\mathrm{molecules}}{{\mathrm{NH}}_{3}\mathrm{sorbed}\,\;\;\mathrm{molecules}\,\;\mathrm{on}\,\;\;\mathrm{the}\,\;\;\mathrm{catalyst}\times \mathrm{time}}$$

The previous formula assumes that NH_3_, with a reactivity similar to that of C_4_H_9_NH_2_ amine, is an appropriate probe molecule to measure the active sites in Ti_3_C_2_.

Product identification was based on GC/MS analysis.

### DFT Calculations

All DFT calculations were carried out with Gaussian 16 software,^52^ using B3LYP as exchange-correlation functional,^53^ def2-SVP basis sets,^54^ and Grimme’s D3 dispersion correction.^55^ Both the energy minimizations and the search for the “saddle points” corresponding to the transition states (TS) were carried out using Berny’s algorithm.^56^ The TSs were confirmed both via a frequency analysis (observing a single negative frequency that corresponds to the reagent product coordinate) and an intrinsic reaction coordinate analysis^57,58^ to further confirm that the stationary point is a true saddle point between both the reactant and product minima.

## Results and Discussion

MXenes were prepared from the MAX phase following the synthetic protocol previously reported by Lipotov et al.^59^ Briefly, the MAX phase was chemically etched by dispersing Ti_3_AlC_2_ in an NH_4_F/HCl solution to etch the Al layer. Afterward, the resulting accordion-like MXene was subjected to ultrasonication for 5 h with DMSO to exfoliate the stacked form into the MXene layers. The MXene layers were observed by high-resolution TEM. Figure 1 shows the high-resolution TEM images of MXene layers with an average lateral size of around 80 nm. When the nanoparticle size in the aqueous suspension was measured by dynamic laser scattering (see below), the apparent nanoparticle size was somewhat higher, with the maximum size distribution around 110 nm, probably due to nanoparticle solvation. Selected area electron diffraction (SAED) reveals a highly crystalline material at the nm length scale, with well-defined bright spots in the SAED pattern of various particles. It has been reported that the SAED pattern of single-crystal Ti_3_C_2_ is a hexagon,^60^ and the fact that we observed more than one would indicate the presence of polycrystalline domains in our sample. The main preferential plane in Ti_3_C_2_ is the (0001) basal plane. This plane is primarily exposed after etching the Al layers from the parent Ti_3_AlC_2_ precursor, which leaves behind a 2D layered structure.

**Figure 1:**
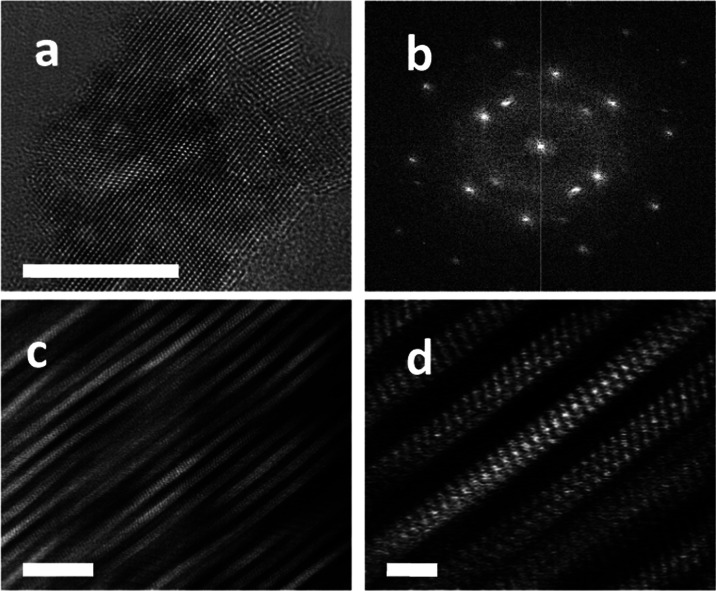
High-resolution TEM images at different magnifications; scale bars: 10 (a), 5 (c), and 1 (d) nm. (b) SAED pattern of Ti_3_C_2_ sheets, revealing their high crystallinity.

AFM is a useful characterization technique for 2D materials that serves to determine the particle thickness with subnanometric resolution. Figure 2 shows the AFM images of the Ti–MXene samples under study in which a thickness distribution between 3 and 9 nm with an average of about 7 nm was determined from the thickness measurement of a statistically relevant number of particles. Interestingly, in contrast to the vertical measurements that can be used to determine particle thickness, frontal AFM images do not provide accurate measurements of particle lateral size when the size of the AFM tip used to obtain the images is not negligible, as in the present case. Information on the lateral particle size was obtained from the TEM images as described previously.

**Figure 2:**
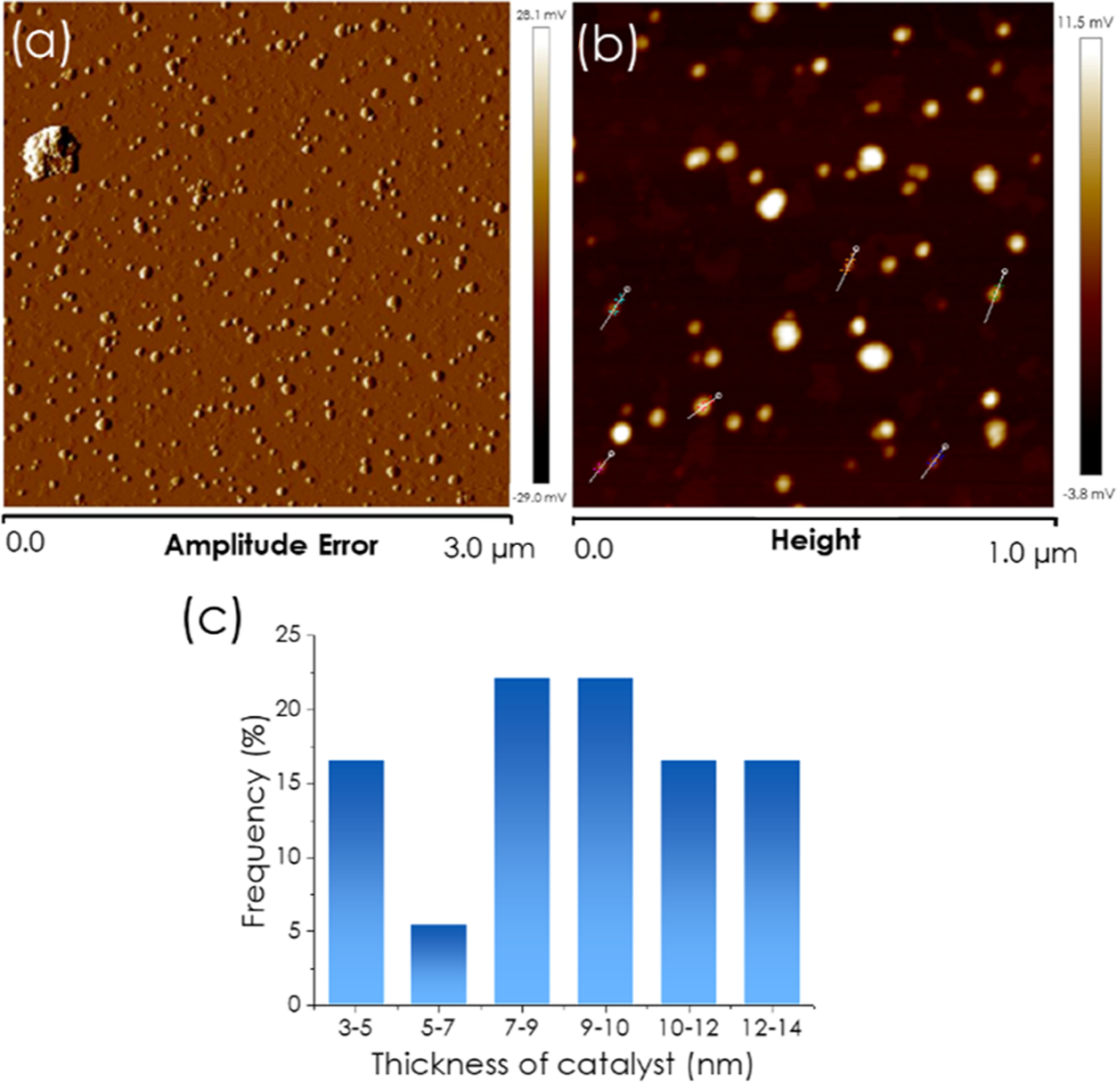
(a, b) Frontal AFM images of Ti_3_C_2_ MXene on an atomically flat mica support at different magnifications; and (c) statistical analysis of thickness distribution measured by AFM for the Ti_3_C_2_ MXene sample used as a catalyst.

The exfoliated Ti–MXene samples were also characterized by XRD. Figure 3a shows a comparison of the XRD patterns of the pristine MAX phase, the resulting accordion-like MXene after Al etching, and the exfoliated MXene layers used as catalysts in the present study. The disappearance of the 2θ 39° peak corresponding to diffraction along the (104) plane reveals the absence of Ti_3_AlC_2_ and confirms the formation of the derived MXene phase. The newly emerged low-angle peak, indexed as the (002) diffraction, is the characteristic peak of unexfoliated, accordion-like Ti_3_C_2_. This accordion-like phase consists of loose stacking of MXene sheets as reported for most MXenes, which implies that the Ti_3_AlC_2_ MAX phase was completely converted to Ti_3_C_2_ MXene flakes. The (002) peak of Ti_3_C_2_ MXene broadens and downshifts significantly toward a lower 2θ angle of 5.94°. The more the (002) characteristic peak shifts to a lower angle, the larger the interlayer spacing of the nanosheets. In the present case, a value of 1.50 nm between the layers was estimated from the XRD 2θ value.

**Figure 3:**
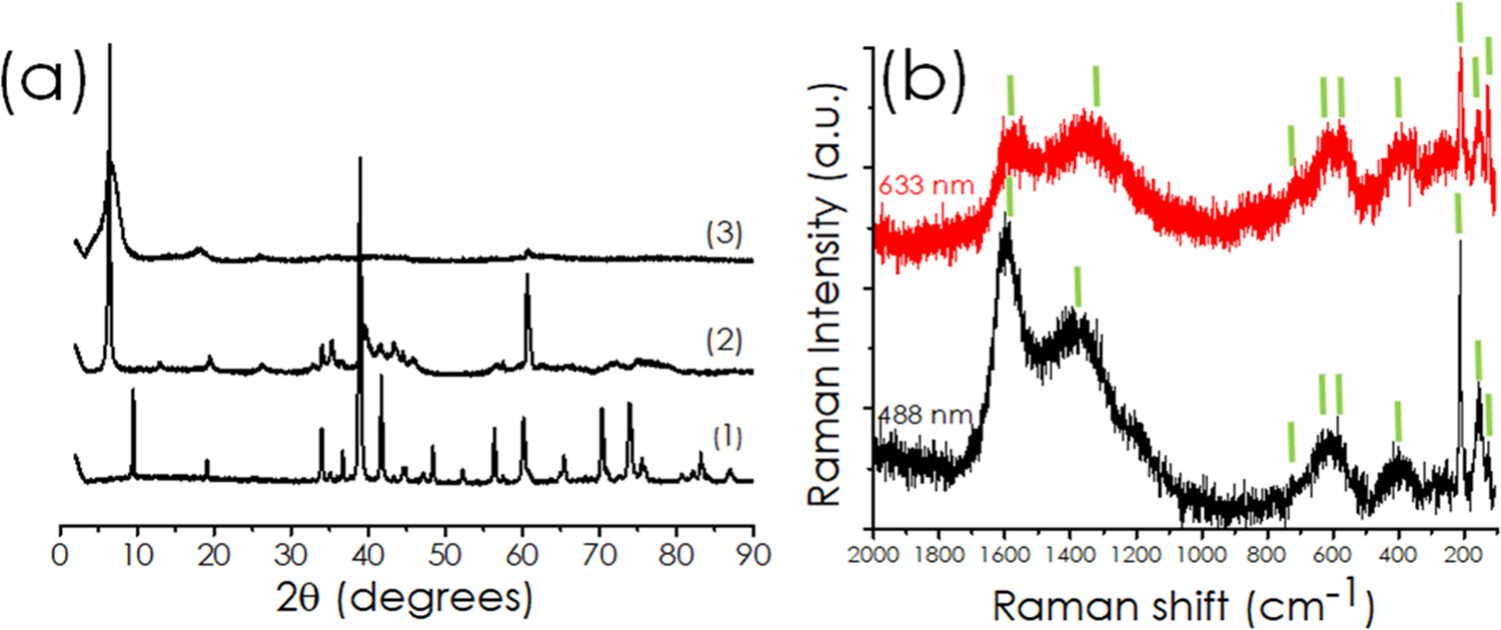
(a) XRD patterns of (1) MAX phase (Ti_3_AlC_2_); (2) Ti_3_AlC_2_ after HCl + NH_4_F etching; (3) delaminated MXene flakes (Ti_3_C_2_); (b) Raman spectra of the Ti_3_C_2_ catalyst recorded at excitation wavelengths of 488 nm (black line) and 633 nm (red line). The green lines indicate the wavenumber values indicated in the text, from left to right: 1599, 1389, 720, 622/621, 584, 380, 213, 154, and 128 cm^–1^, respectively.

The Raman spectra of the investigated Ti_3_C_2_ present the expected bands corresponding to the various Ti–MXene vibration modes due to carbide layers, Ti–C vibrations, and Ti surface functional groups. The characteristic Raman peaks of Ti_3_C_2_ are indicated by the green lines in Figure 3b. Thus, the in-plane Ti_3_C_2_ *E*_g_ vibration modes appear at 154 and 621 cm^–1^, while the in-plane Ti_3_C_2_O_2_ Eg vibration modes are recorded at 380 and 584 cm^–1^. For the Ti_3_C_2_O_2_ *A*_1g_ vibration mode, the Raman shift is 720 cm^–1^ and the in-plane Ti_3_C_2_(OH)_2_ *E*_g_ vibration modes are recorded at 128 and 622 cm^–1^. Finally, the Ti_3_C_2_(OH)_2_ *A*_1g_ mode appears at 213 cm^–1^.^61^ The peaks at 1389 and 1599 cm^–1^ correspond to the D (peak attributable to defects) and G modes of the carbon layer.^62^

Figure 4a shows the diffuse reflectance UV–vis–NIR (DR UV–vis) absorption spectrum of the investigated Ti_3_C_2_ MXene. The DR UV–vis absorption spectrum of Ti_3_C_2_ presents a UV band in the region of 250–350 nm and a broad tail with onset at about 500 nm.^63,64^ In addition, the zeta potential of the Ti_3_C_2_ MXene suspension at neutral pH was measured, giving a value of −45 mV, which corresponds to a persistent suspension due to the Coulombic repulsion of highly charged negative sheets (Figure 4b). Dynamic laser scattering measurements establish a broad particle size distribution of the suspended particles, centered at about 110 nm (Supporting Information Raw Data Section). This apparent average size is larger than that previously reported from TEM measurements of about 80 nm since the values of laser scattering correspond to the solvated Ti–MXene particles in comparison to dry particles observed by electron microscopy. The composition of Ti_3_C_2_ MXene was confirmed by XPS analysis. Survey XP spectra show the presence of Ti, C, O, and F. The peak corresponding to Al was not detected in the survey XP spectrum, confirming that the Al layers of the Ti_3_AlC_2_ precursor were removed with NH_4_F/HCl etching treatment. High-resolution XPS spectra corresponding to the C 1s, O 1s, and Ti 2p core levels of Ti_3_C_2_ are presented in Figure 5. The C 1s peak can be deconvoluted into three peaks, namely, graphitic C at 284.6 eV, C bonded to Ti, shifted to lower binding energies (284.0 eV), and C bonded to O, appearing at higher binding energies (288.0 eV). On the other hand, Ti 2p was deconvoluted into three peaks corresponding to Ti bonded to C (454.4 eV), to O (458.0 eV), and to F (463.0 eV). The analysis of the O 1s peak supports the conclusion based on the analysis of the XPS C 1s and Ti 2p levels. In this way, the two components of O 1s appearing at 529.0 and 531.0 eV can be assigned to surface terminal O atoms connected to Ti,^65^ while the third at 532.5 eV to O=C–OH.^66^ All the previous XPS data are in good agreement with the literature.^67^ To further confirm the structure and quality of the Ti_3_C_2_ sample under study, X-ray absorption spectroscopy measurements were carried out. Figure 5 shows the normalized Ti K-edge X-ray absorption near-edge structure (XANES) spectra of Ti_3_C_2_ MXene and the references Ti foil, TiC, TiO_2_, and Ti_2_O_3_. The edge energy of Ti_3_C_2_ is close to that of TiC, which is between the energies of the Ti foil and TiO_2_, indicating its carbide nature with a valence state lower than +4. The κ-pre-edge is dominated by C­(2p)-Ti­(3d) hybridization, while the main edge is dominated by Ti 1s → 4p excitation.^68,69^ Figure 5 also shows the Ti K-edge Fourier transform extended X-ray absorption fine structure (FT-EXAFS) of the Ti_3_C_2_ MXene sample and the corresponding reference compounds. The Fourier transform of the EXAFS spectra of Ti_3_C_2_ MXene shows first-shell scattering (Ti–C/O) similar to that of TiC but with a second-shell scattering (Ti–C–Ti) lower than that of TiC, which is consistent with the reduced dimensionality of Ti_3_C_2_ MXene in which there should be a high proportion of surface Ti atoms. The EXAFS fitting curves and the fitting results of Ti are shown in Figure S1 and Table S1. All these spectra are in agreement with the reported data for other Ti_3_C_2_ samples, indicating that the results presented here should also be valid for other Ti_3_C_2_ samples.^70^

**Figure 4:**
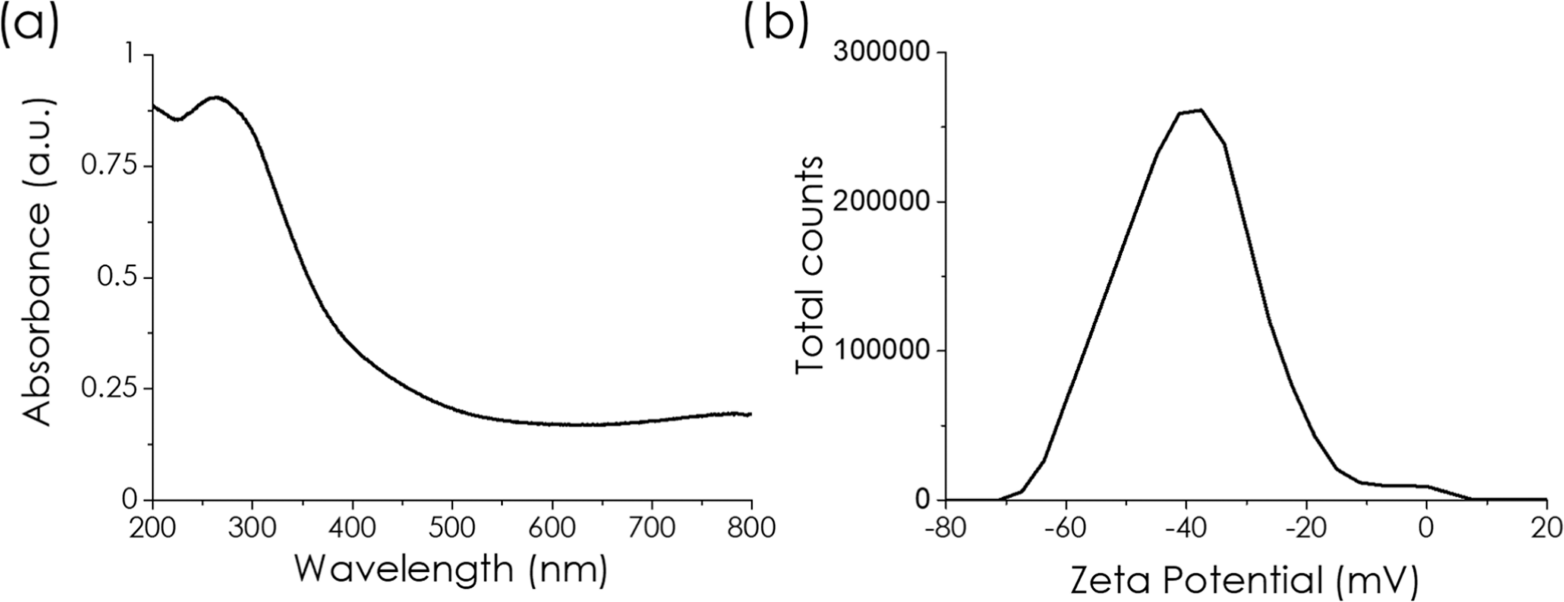
(a) UV–vis spectra of Ti_3_C_2_ MXene and (b) ζ-potential of the Ti_3_C_2_ MXene suspension.

**Figure 5:**
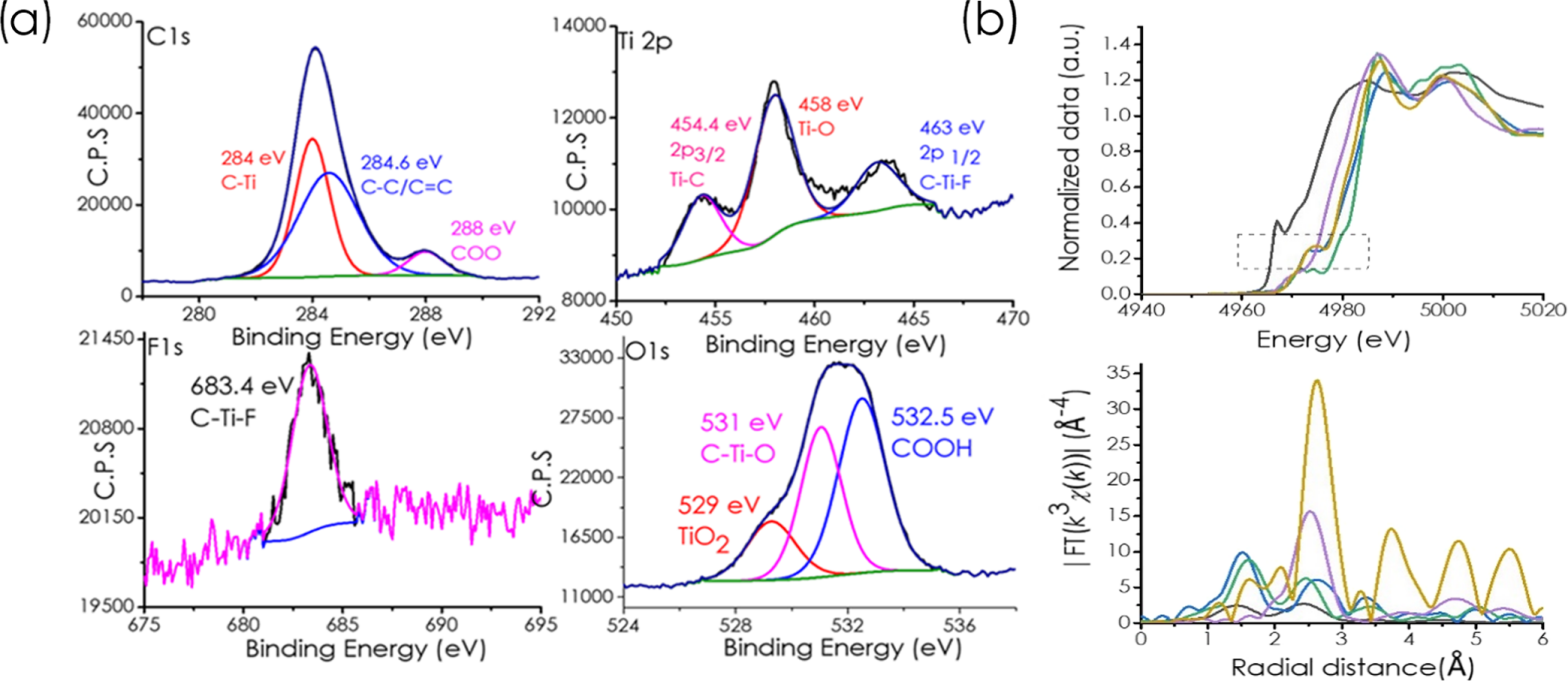
(a) High-resolution XP spectra corresponding to the C 1s, O 1s, and Ti 2p levels for Ti_3_C_2_ MXene, as well as the best fit to individual components as indicated in the plots. (b) Top: Ti K-edge XANES highlights the important region in the dotted square; bottom: Ti K-edge R-space EXAFS spectra of Ti_3_C_2_ MXene (blue) with references of Ti foil (black), Ti_2_O_3_ (purple), TiO_2_ (green), and TiC (khaki).

Table 1 presents the results of the pulsed H_2_ chemisorption and H_2_-_,_ CO_2_-, and NH_3_-TPD. The interaction of H_2_ with MXenes can occur via physisorption, Kubas-type interaction with the transition metal resulting in an elongation of the H–H bond, but without dissociation, and dissociative H_2_ chemisorption that usually results in H spill over the whole surface.^71^ The combination of these three interactions of H_2_ with the MXene surface can explain the much higher amount of H_2_ sorption compared with the amount measured by H_2_-TPD, with the difference corresponding to weakly physisorbed H_2_. In H_2_-TPD, the presence of two broad peaks at 210 and 301 °C would indicate that there are two surface adsorption sites on the 2D MXene structure, probably associated with terminal group vacancies, structural defects, and peripheral atoms.^72^

**Table 1:** Summary of the Pulsed H_2_ Sorption and H_2_-, CO_2_- and NH_3_-TPD Data

experiment	temperature (^o^C)	amount (mmol/g)
pulsed H_2_ adsorption	RT	27.95
H_2_-TPD	210	0.31
	301	0.70
	total	1.01
CO_2_-TPD	318	0.014
NH_3_-TPD	316	0.004

Both the acidity and basicity of this material were rather small (below 0.015 chemisorbed probe mmol/g, Table 1), particularly considering the higher amount of sorbed H_2_ molecules, with a threefold higher intensity for CO_2_ (basic sites) than for NH_3_ (acid sites). It is important to note here that beyond the acidity measurement, information from NH_3_ titration of Ti_3_C_2_ can also be taken as a quantitative determination of the maximum density of sites that can interact with amines and, therefore, an estimation of the maximum population of sites that could participate in hydroamination catalysis.

Since the reaction mechanism requires amine adsorption as the first step (see below), NH_3_ titration measurements can be interpreted as an appropriate technique to determine the maximum number of hydroamination sites present on Ti_3_C_2_. It is also likely that NH_3_ overestimates the density of active sites on Ti_3_C_2_ since NH_3_ is smaller and could be more basic than the amines used as reagents in hydroamination, particularly substituted aromatic amines.

## Catalytic Activity

As mentioned earlier, the hydroamination of alkynes is an important synthetic reaction for the preparation of imines, secondary amines, and nitrogen heterocycles in which acid catalysis is frequently inefficient due to amine protonation.^73^ Acid catalyst deactivation is particularly notable for more basic aliphatic amines. Titanium complexes have been reported as molecular catalysts to promote hydroamination.^47,74,75^ However, to the best of our knowledge, there are no heterogeneous titanium catalysts able to promote the reaction, and the known heterogeneous catalysts reported so far are very few and frequently contain Au^44,76^ and other noble metals.^77,78^ The main reason for the low hydroamination activity of Ti solids is the high oxophilicity of Ti that, by reaction with moisture, forms stable titanium oxides that are devoid of catalytic activity. Considering the advantages of heterogeneous catalysis in terms of the easy separation of the reaction mixture and the possibility of reuse, it is important to determine whether Ti_3_C_2_ MXene can act as a hydroamination catalyst. From the preliminary NH_3_-TPD data, it could be predicted that, if any, the Ti_3_C_2_ catalytic activity should be very low due to the low site density. Initial catalytic studies were carried out using a more basic aliphatic amine.

### Hydroamination of 1-Hexyne with *n*-Butylamine

A blank control shows that no products are formed under the reaction conditions in the absence of a catalyst or when TiO_2_ is used as a solid catalyst. In contrast, it was found that Ti_3_C_2_ MXene promotes the electrophilic addition of *n-*butylamine to 1-hexyne, forming predominantly one of the two possible *regio*isomers depending on the position of the *n-*butylamine attack (Scheme 1). Working under an N_2_ atmosphere, the selectivity of the reaction was 99:1 in favor of the anti-Markovnikov product.

**Scheme 1:**
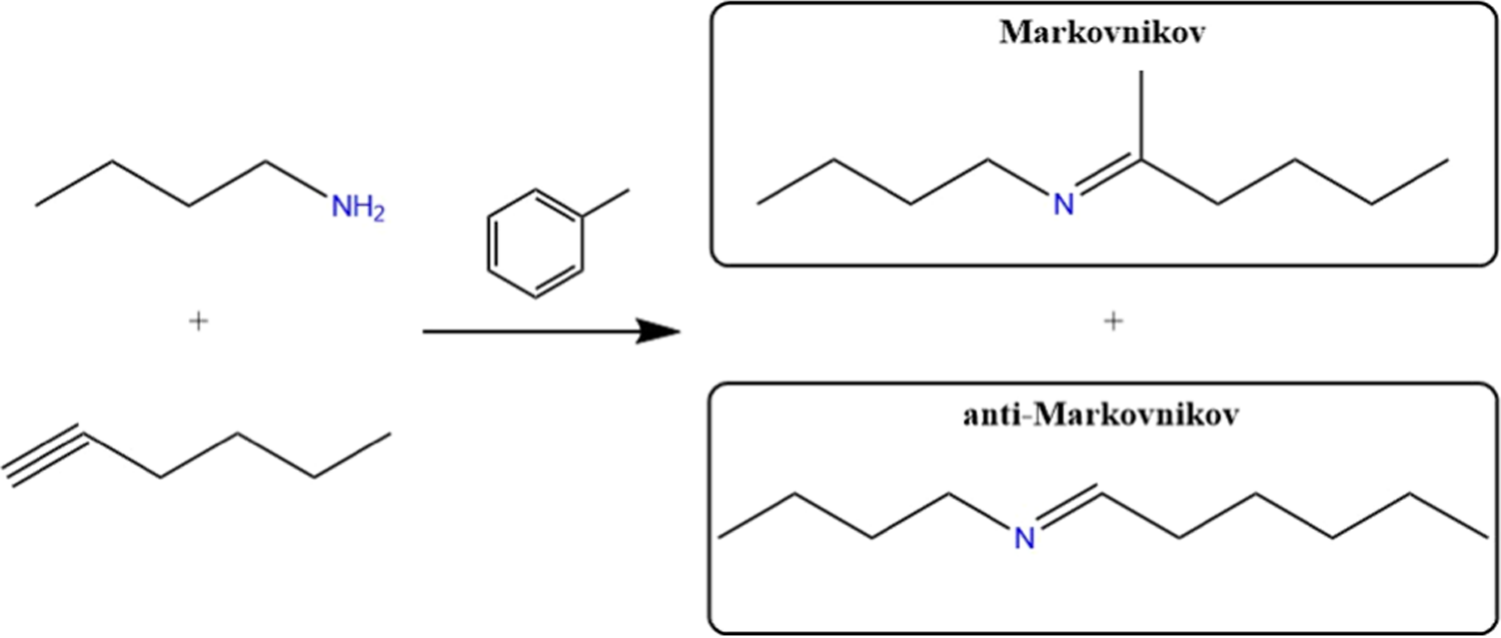
Electrophilic Addition of *n*-Butylamine to 1-Hexyne Leading to Two Possible Regioisomers (Markovnikov and Anti-Markovnikov Products) Depending on the Position of the n-Butylamine Attack

Figure 6 shows the results of the hydroamination of 1-hexyne with *n*-butylamine on the Ti_3_C_2_ catalyst as a function of the reaction temperature. As can be seen in Figure 6, the conversion increased significantly in the temperature range from 140 to 160 °C and with the reaction time but without influence on the product selectivity, which was constant at 99:1 for the anti and Markovnikov isomers, respectively. This indicates that both *regio*isomers are the primary products that are stable under the reaction conditions. For the quantification of active sites, the similarity between NH_3_ adsorption and organic amine adsorption was considered. It is proposed that the active sites interacting with the amine correspond to a fraction of the Lewis acid sites titrated by NH_3_-TPD in which Ti atoms interact with NH_3_ and, therefore, possibly also with amines. In this way, knowing that the NH_3_ molecules adsorbed per gram of Ti_3_C_2_ as shown in Table 1, the minimum limit for the TOF values can be calculated. It should be noted that compared to NH_3_, alkylamines have larger molecular sizes that could preclude their interaction with some Ti atoms on the Ti_3_C_2_ sheet due to steric encumbrance. Therefore, the TOF values based on NH_3_ titration should be taken as the lowest TOF estimate since certain Ti atoms with steric restrictions can adsorb NH_3_ but are unable to adsorb larger molecules. Therefore, the number of active sites could be much lower. As shown in Figure 6, the TOF values measured for conversions ≥5% increased with temperature, while remaining unchanged from 24 to 48 h (Figure 6). This indicates that the sites initially present in the fresh catalyst remain active during the reaction without undergoing deactivation. Otherwise, the initial TOF values should be higher than the TOF values calculated at longer reaction times when deactivation could have occurred to some degree. The maximum yield of *N*-hexylidene-1-butylamine under reaction conditions of 160 °C for 48 h was 34%. To determine the influence of pressure on hydroamination, an additional reaction was carried out using the same amounts of reagents and Ti_3_C_2_ catalyst with the same toluene volume; however, the reactor was pressurized with N_2_ at 10 bar at ambient temperature. Then, the reaction was carried out at 160 °C for 24 h, whereby the anti-Markovnikov hydroamination product was formed in 28% yield with a TON of 583 h^–1^.

**Figure 6:**
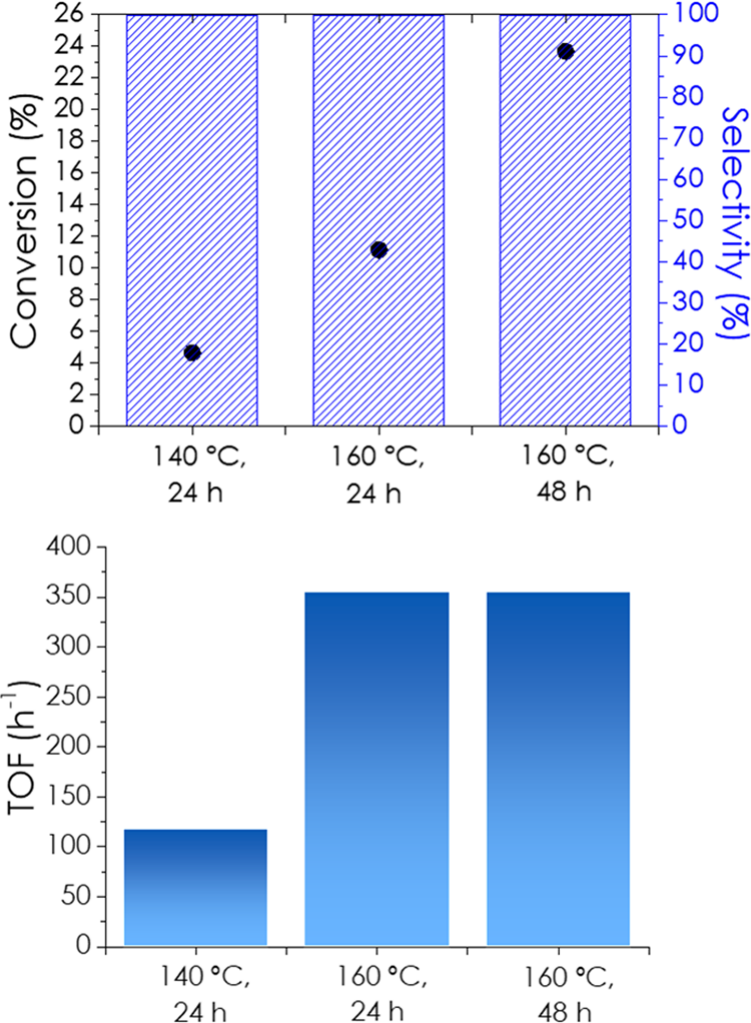
Hydroamination of 1-hexyne with *n-*butylamine under various conditions. Top: variation in 1-hexyne conversion and selectivity to the anti-Markovnikov imine with temperature and time; bottom: TOF values at different times and temperatures. Reaction conditions: 5 mg Ti_3_C_2_, 2 mmol 1-hexyne, 1 mmol *n*-butylamine, 2 mL toluene, and N_2_ atmosphere.

The general catalytic activity of the Ti MXenes was confirmed by using Ti_2_C as the catalyst. It is known that Ti_2_C undergoes spontaneous oxidation in an aqueous suspension in the presence of air. Under these conditions, the black Ti_2_C material was oxidized to white TiO_2_. It has been reported that this oxidation can be avoided by storing the aqueous Ti_2_C suspension under Ar, avoiding exposure to O_2_.^79^ A control using the white material as the hydroamination catalyst did not result in the formation of the hydroamination product, indicating that the TiO_2_ derived from Ti_2_C is totally inactive as a catalyst. In contrast, black Ti_2_C catalyzed the reaction of 1-butylamine and 1-hexyne giving 9.0% of the hydroamination product after 48 h under a N_2_ pressure of 10 bar at 200 °C.

Thus, it is important to rank the catalytic activity of Ti_3_C_2_ compared with other heterogeneous or homogeneous catalysts. Table 2 provides a comparison of the TOF values obtained for Ti_3_C_2_ with those reported in the literature for other catalysts. As can be seen in Table 2, the TOF values of Ti_3_C_2_ (on the order of 10^2^ h^–1^) were, indeed, very high with respect to the values reported for hydroamination, which is attributed to the adequate acid strength of the Ti_3_C_2_ sites and the possible cooperation of two or more Ti atoms in the reaction mechanism. It should be noted that too strong acidity is detrimental due to the inactivation of the amine reagent. Thus, the electron density provided by the negative carbide ions of the underlayer in Ti_3_C_2_ seems to tune the Lewis acid on Ti. In addition, proper control of the surface terminal groups (−F, −O–, and −OH) could also result in further optimization of the Ti_3_C_2_ catalytic activity. It is worth noting that a control using TiO_2_ as the catalyst does not lead to measurable product formation, showing the distinctive behavior of Ti carbide compared to an oxide.

**Table 2:** Comparison of the Catalytic Activity of Ti_3_C_2_ with those of Other Related Homogeneous and Heterogeneous Hydroamination Catalysts

catalyst	reagents	conditions	TOF (h^–1^)	ref
Ind_2_TiMe_2_ (Ind: indenyl)	*n*-octyne toluene	catalyst 5 mol %, 105 °C, 1 h	20^a^	^83^
bisamidate bis(diethylamine)titanium	*n*-hexyne benzylamine	catalyst 5 mol %, 65 °C, 24 h	0.80^a^	^84^
DPymTi(NMe)_2_ (DPym:dipirrolylmethane)	*n*-hexyne aniline	catalyst 5 mol %, 25 °C, 5 min	120^b^	^85^
V(NMe_2_)_4_	*n*-hexyne aniline	catalyst 10 mol %, 80 °C, 20 h	0.30^a^ ^,^ ^b^	^75^
titanocene.TMSC≡CTMS (TMS: trimethylsilyl)	*n*-hexyne aniline	catalyst 5 mol %, 100 °C, 24 h	0.78	^50^
Cu^+^/β zeolite^d^	phenylacetylene aniline	catalyst 0.1 Cu mol %, 111 °C, 8 h	165^b^	^48^
Cu–K-10 (K-10: montmorillonite)	*n*-hexyne aniline	catalyst 0.1 Cu mol %, 80 °C, 20 h	0.7^a^ ^,^ ^b^	^86^
Au/ZnO	phenylacetylene aniline	catalyst 0.5 Au mol %, 100 °C, 7 h	20^b^	^87^
Au–chitosan–SiO_2_	*n*-hexyne aniline	catalyst 1.1 Au mol %, 100 °C, 2 h	45^a^ ^,^ ^b^ ^c^	^88^
Ti_3_C_2_ MXene	*n*-hexyne butylamine	catalyst 5 mg, 140 °C, 24 h	350	this work
Ti_3_C_2_ MXene	*n*-hexyne aniline	catalyst 5 mg, 160 °C, 24 h	100	this work

a Estimated from the reported data.

b Markovnikov as the main regioisomer

c Ketone by imine hydrolysis was formed together with imine in 45%

d Cu^+^ ions are unstable and tend to undergo spontaneous oxidation.

It is worth mentioning here that the adequate acid/base strength of Ti sites should be a consequence of the work function of the Ti_3_C_2_ material, which is a collective property of MXenes, as opposed to the local characteristics of each site. Experimental measurements have shown that the electronic properties of Ti_3_C_2_ MXene obtained by F etching can vary as much as 1 eV, depending on the exact surface functional group distribution among −F, −O–, and −OH, vacancies.^22^ Therefore, it can be concluded that the observed hydroamination activity could be further fine-tuned by adjusting the collective MXene properties such as the work function, nature of the surface functional groups, number of the carbide/nitride layers, and exfoliation degree.

Importantly, the Ti_3_C_2_ catalyst can be easily recovered from the reaction mixture, washed with toluene, and reused. No change in the catalytic activity of Ti_3_C_2_ was observed after four consecutive cycles (see Supporting Information, Figure S2). This performance is remarkable considering the low density of the active sites and the ability of alkylamines to act as poisons or promoters of oligomerization of homogeneous catalysts. It also seems to rule out coke deposition under the reaction conditions, as further confirmed by Raman spectroscopy in which no increase in the intensity of the 1590 and 1350 cm^–1^ bands characteristic of coke was observed for the Ti_3_C_2_ sample after its use as a catalyst (see Supporting Information). Further characterization of the four timnes used Ti_3_C_2_ sample showed that crystallinity was maintained, as confirmed by XRD and selected area electron diffraction in TEM, while AFM measurements showed that the thickness of the layers was about 4 nm without apparent stacking or agglomeration (see Figure S2).

The hot filtration test indicates that the reaction stops upon removal of the Ti_3_C_2_ catalyst (Figure S3), indicating that the reaction is truly heterogeneous, occurs on the solid catalyst, and is not due to the leaching of Ti from the solid to the liquid phase. Analysis of the liquid phase after the reaction showed that the amount of Ti in the supernatant was lower than 1% of the total amount of Ti added to the Ti_3_C_2_ catalyst. Characterization of MXene after the reaction by TEM shows that the morphology of the particles and their crystallinity were maintained under the reaction conditions. These data confirm the absence of deactivation and amine poisoning under the reaction conditions.

### Scope of Catalytic Hydroamination by Ti_3_C_2_

Considering that aliphatic primary amines are more reactive than aromatic amines and that aryl-substituted imines are important synthetic intermediates, the possible catalytic activity of Ti_3_C_2_ was also tested for this type of amine. Under an N_2_ atmosphere, the catalytic hydroamination using aniline followed the same trend as that determined for the aliphatic amine, namely, an almost total selectivity (99:1) in favor of the (1*E*)-*N*-phenylhexan-1-imine isomer and an increase of the conversion with temperature and reaction time (Figure 7). Other aromatic amines, including 4-ethylaniline and 2,6- and 3,5-dimethylanilines, were also tested, and the TOF values measured after 24 or 48 h are also shown in Figure 7. The maximum yield of *N*-hexylidene-4-ethylaniline after 48 h was 12%. Hydroamination of 1-hexyne failed to form products in the case of 4-aminophenol and 4-nitroaniline, indicating that this reaction is highly sensitive to the influence of the electron-donating or -withdrawing nature of substituents on the aromatic ring, as observed in other cases, such in the use of Pd­(II) complexes as homogeneous hydroamination catalysts of aromatic amines.^80^

**Figure 7:**
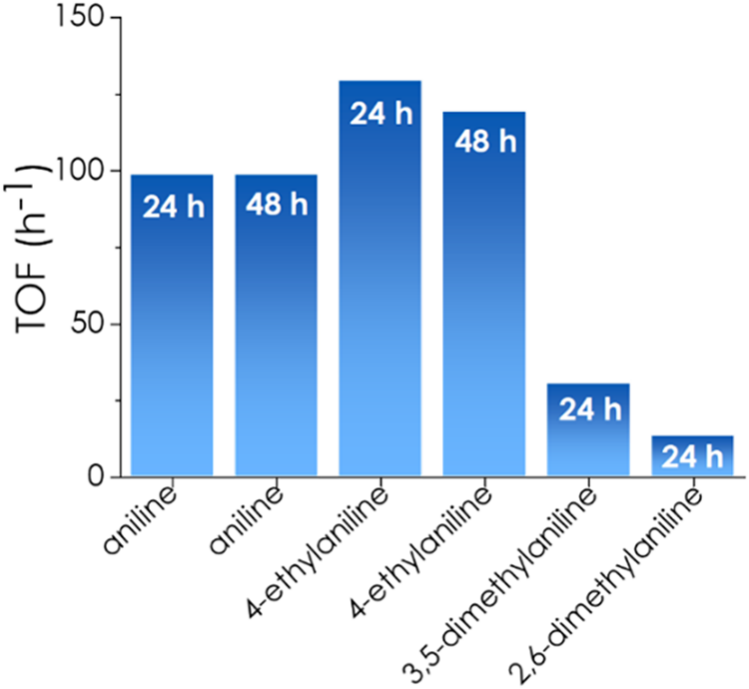
TOF values for hydroamination of 1-hexyne with aromatic amines. Reaction conditions: 1 mmol of 1-hexyne, 2 mmol of aromatic amine, 2 mL of toluene as a solvent, purged with N_2_, Ti_3_C_2_ 5 mg, 160 °C. The time at which the TOF values were calculated is shown in the plot.

The reactivity of aromatic amines is about 2 to 10 times lower than that of aliphatic *n-*butylamine and appears to depend on the electronic density and steric encumbrance of the amino group at the aromatic ring. On the one hand, this amine reactivity order indicates that Ti_3_C_2_ as a heterogeneous catalyst performs as expected for a mild Lewis acid site; on the other hand, the operation of notable steric effects in the reaction, thereby explaining the higher reactivity of aniline and *para-*substituted anilines compared to the *ortho* and disubstituted analogues. 1-Phenylacetylene with a conjugated C≡C triple bond also reacts regioselectively with anilines giving the corresponding 2-phenyletane N-arylimines (Table S2). In this case, the TOF values were similar to those measured for 1-hexyne with anilines, indicating that the electronic density of the C≡C bond compensates for the higher steric hindrance caused by the phenyl ring.

Further studies on the reaction scope showed that Ti_3_C_2_ was unable to promote the hydroamination of internal −C≡C– triple bonds under the present reaction conditions (toluene at 160 °C). Thus, all of the hydroamination reactions tested with 4-octyne and 1-phenyl-1-hexyne failed (see Table S2). The failure of hydroamination using internal alkynes can be understood by considering the higher steric demand required for the reaction of internal alkynes and their lower reactivity.

Importantly, switching the reaction atmosphere from N_2_ to air resulted in a change in reactivity, as observed with the expected *N*-phenylhexanimines, the formation of dodeca-5,7-diyne, and diazenes (Scheme 2). The homocoupling of both alkynes^81^ and aromatic amines^82^ requires the presence of aerobic oxygen as an oxidizing reagent. The formation of the oxidation products derived from homocoupling was observed as well in the reactions of 1-hexyne with 3,5-dimethylaniline, 2.6-dimethylaniline, and 4-ethylaniline for which the conversion, selectivity, and TOF values depended on the substrate. Figures S4–S7 show the product distribution in air, and the Supporting Information provides the mass spectra of the observed oxidative coupling products. Under an air atmosphere, the selectivity to dodeca-5,7-diyne decreased in the following order: 2,6-dimethylaniline > 3,5-dimethylaniline > 4-ethylaniline > aniline, which correlates well with the relative reactivity of the aromatic amine toward hydroamination; more reactive aromatic amines formed fewer byproducts in the presence of oxygen. These catalytic data clearly indicate that oxidative homocoupling is a competing reaction that occurs when hydroamination is disfavored and oxygen is present. For this reason, oxidation byproducts were not observed in the reaction of the more reactive *n-*butylamine with 1-hexyne. Although aerobic oxidations are an important class of organic reactions largely performed for the production of bulk chemicals and it appears Ti_3_C_2_ also has an interesting activity worth exploring to promote this liquid-phase oxidation, its study will be reported separately. Therefore, in the case of hydroamination, it is recommended to exclude air during the reaction.

**Scheme 2:**
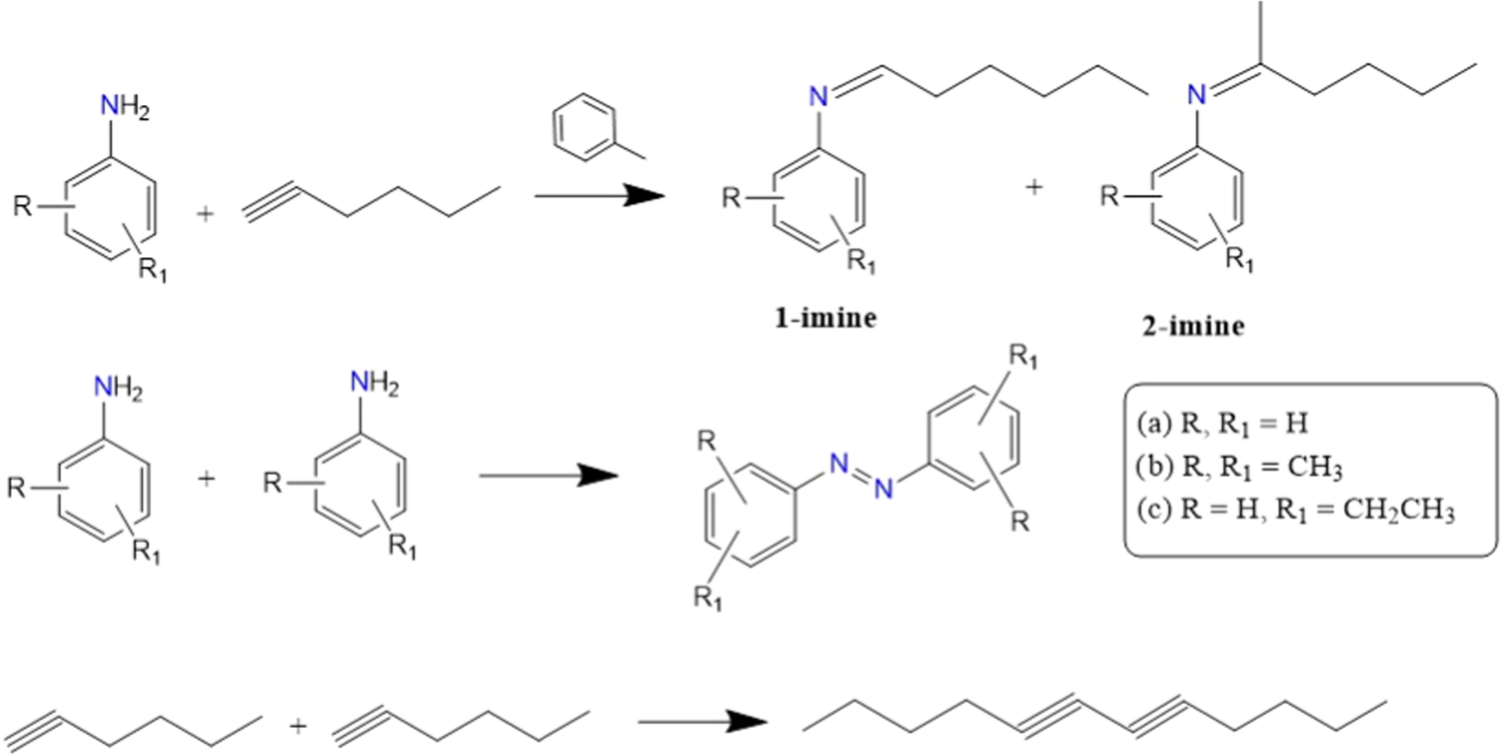
Competition between Hydroamination and Oxidative Homocoupling in the Reaction of 1-Hexyne and Aromatic Amines when Oxygen is Present

### Comparative Catalytic Activity of Ti MXene as a Hydroamination Catalyst

Table 2 provides a summary of some of the most efficient reported hydroamination catalysts taken from the several comprehensive reviews existing in the literature covering the field of hydroamination catalysis, both homogeneous and heterogeneous.^41,48,49^ As can be seen in Table 2, the results obtained in the present study rank Ti_3_C_2_ as one of the best solid catalysts for the hydroamination reaction reported so far. As discussed in the Introduction section, the most studied hydroamination catalysts are soluble metal complexes, frequently early transition metals of group IV of the Periodic Table. These soluble complexes exhibit generally higher TOF values than heterogeneous catalysts.^41^ In comparison, heterogeneous catalysts can be recovered from the reaction mixture by filtration and have advantages in terms of catalyst recycling and the possibility of developing continuous-flow processes.

Comparison of the catalyst performance based on the reported data should always be taken cautiously because some of the activity values, particularly TOFs, were reported under different experimental conditions, sometimes far from the optimal ones, or measured at different conversions (see also footnote “a” in Table 2). In any case, the data in Table 2 indicate that Ti_3_C_2_ MXene is an excellent hydroamination solid catalyst with complete anti-Markovnikov *regio*selectivity, particularly considering that the TOF values of Ti_3_C_2_ are stable with respect to the reaction time and substrate conversion and that the material can be reused without deactivation in consecutive runs. Even with the required caution, this performance ranks Ti_3_C_2_ as the best choice in the current state of the art.

Besides activity, product selectivity, and stability, another important consideration regarding the potential applicability of a catalyst is its cost. The costs of Ti_3_C_2_ MXene based on the preparation steps and allocating some expenses for a minimal batch characterization gave a value of $12.20 per gram.^89^ However, considering the vast potential applications that are envisioned for MXenes, particularly in the field of energy storage devices, scale-up estimations have considered an achievable cost of Ti_3_C_2_ for bulk sales of 5 cents/g.^90^

### Intramolecular Cyclization

Intramolecular hydroamination can afford aromatic heterocycles with indole or quinoline structures, many of which exhibit remarkable biological activity.^91,92^ To show the general activity of Ti_3_C_2_ MXene as a hydroamination catalyst, intramolecular cyclization of 2-(phenylethynyl)­aniline and 2-(phenylethynyl)­pyridin-3-amine was performed. The results are summarized in Table 3. As can be seen there, in the two cases, a single product with the structure of indol or azaindole, resulting from the 5*-endo-dig* ring closure of the starting material, was obtained. Control experiments in the absence of a catalyst showed negligible substrate conversion under the reaction conditions, with small amounts of (aza)­indol products. In the presence of Ti_3_C_2_ as a catalyst, the yield increased with temperature, either 150 or 200 °C, and was somewhat higher for pyridin-3-amine in comparison with the aniline analogue, particularly at the lowest temperature, reflecting the electron-withdrawing effect of the pyridine ring polarizing the C≡C triple bond.

**Table 3:**
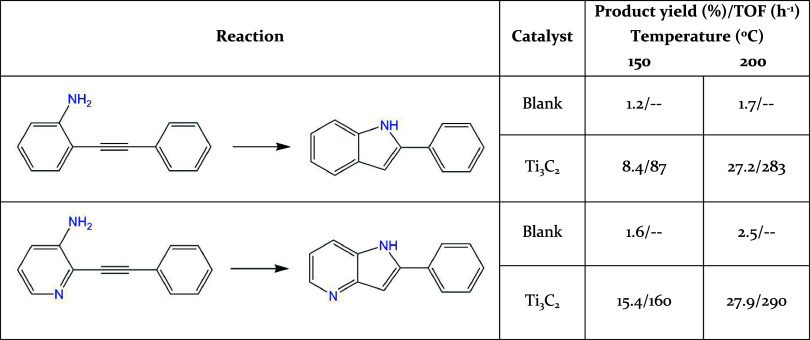
Results of Intramolecular Hydroamination Catalyzed by Ti_3_C_2_ ^a^

a Reaction conditions: substrate: 0.5 mmol, solvent: 2 mL of 1,4-dioxane, catalyst: 5 mg, time: 24 h.

### Hydroamination Reaction Mechanism

The formation of both Markovnikov and anti-Markovnikov hydroamination products has been reported using molecular metal complexes as catalysts.^50,93^ Frequently, selectivity toward a single regioisomer has been observed. All the proposed mechanisms involving group IV metals as active sites have in common the formation of key intermediates of a four-member ring with the active metal site being one of the atoms, the N of the amine, and the two carbon atoms of the C≡C triple bond completing the cycle. In the case of Ti atoms, this four-member ring intermediate is proposed to arise from the [2 + 2] cycloaddition of the alkyne and the Ti=N bond, the latter arising from the faster reaction of the amine and the Ti site. According to Beller and co-workers, in the case of titanocene complexes as hydroamination catalysts,^50^ the regioselectivity of the final product is controlled by the steric encumbrance experienced by the alkyne as it approaches the Ti=N intermediate (Scheme 3). Titanocenes can be considered to have some structural resemblance to Ti_3_C_2_ MXene in the sense that one or two aromatic C rings (cyclopentadienes) form a strong π complex with a Ti ion that has exchangeable terminal groups, completing the coordination sphere. Therefore, it is proposed here that the same steric factor operating in molecular titanocenes is responsible for the observed anti-Markovnikov regioselectivity in the case of Ti_3_C_2_ MXene (Scheme 3). The less congested *regio*isomer, corresponding in this case to the anti-Markovnikov product, is also formed in the present case with a very high preference with respect to the thermodynamically more stable isomer that would be formed from the more congested transition state. This proposal was supported by DFT calculations of the models described below.

**Scheme 3:**
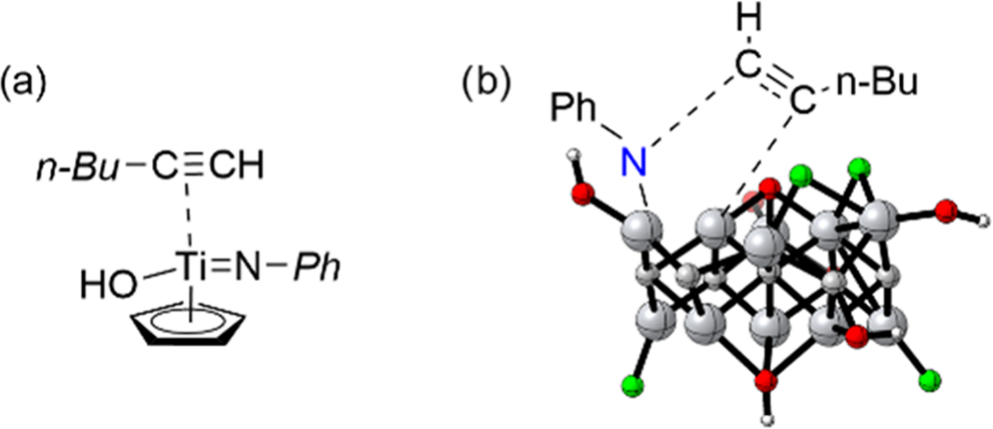
Proposal to Rationalize the Anti-Markovnikov Selectivity Based on the Steric Congestion (Ph and n-Butyl Groups as far as Possible) Accepted in Homogeneous Titanocene Complexes (a, ref 50) and that it is also Proposed to Operate in Heterogeneous Ti_3_C_2_ Catalysis (b, this work)

#### In Situ Raman and ^13^C NMR Spectroscopy

To gain insight into the operating mechanism in the presence of Ti_3_C_2_, the reagents were adsorbed sequentially on the Ti_3_C_2_ catalyst, and their interaction with Ti_3_C_2_ was monitored by Raman spectroscopy using two different lasers operating at 488 or 785 nm wavelengths. Adsorption was carried out by stirring a suspension of Ti_3_C_2_ in a toluene solution of *n*-butylamine at 50 °C or aromatic amines at 100 °C for 30 min, after which the solid was recovered, washed with fresh toluene to remove loosely bound amines, and dried. For alkyne adsorption, the Ti_3_C_2_ solid that had previously undergone amine adsorption, likely forming a Ti–N intermediate, was used as the starting sample. The sample was stirred in a toluene solution of 1-hexyne at 50 °C for 30 min. The subsequent washing and drying steps were performed in the same manner as those used for amine adsorption. Using a 488 nm laser for excitation, it was observed that the characteristic low-frequency vibration band corresponding to Ti_3_C_2_O_2_ *E*_g_ underwent significant changes upon amine adsorption up to 20 cm^–1^ redshift, while the other peaks remained unchanged (see Figure S8 in Supporting Information). The fact that the specific Ti_3_C_2_O_2_ vibration undergoes shifts while other bands remain at the same position indicates that amine adsorption takes place at certain sites influencing this specific surface functional group but not others. Interestingly, the subsequent addition of alkyne produced clear, measurable shifts in the Raman frequency of the vibration band that was previously modified by amine adsorption. The fact that not all the vibrations shift upon the addition of the alkyne after the addition of the amine is again considered a sign that Raman spectroscopy reports the participation of certain functional groups of the Ti_3_C_2_ catalyst.

Raman spectroscopy using 785 nm laser excitation provided further information (see Figure S10 in Supporting Information). Upon the adsorption of *n*-butylamine, three new peaks appeared at 505, 270, and 260 cm^–1^. Based on the literature data,^95^ these new peaks can be attributed to the in-plane (505 cm^–1^) and out-of-plane (270 and 260 cm^–1^) vibrations of the −OH groups, indicating that these groups appear on the Ti_3_C_2_ surface upon adsorption of *n-*butylamine. This indicates that the adsorption of *n-*butylamine results in the generation of surface −OH groups by proton transfer from the amine to the surface −O– atoms (step prior to cycloaddition in Scheme 4). Since amines are weaker Brönsted acids than −O– groups, this proton transfer can only occur if the amine interacts with Ti atoms as Lewis acids. Subsequent addition of 1-hexyne restores the initial medium-frequency Raman spectrum of Ti_3_C_2_ to its initial state, indicating that −OH groups disappear, which requires the reaction of the alkyne with the Ti=N adduct (step indicated as proton transfer in Scheme 4). Reversing the order of reagent addition, 1-hexyne and then 1-butylamine, does not result in any change in the Raman spectrum, again reinforcing the interaction of MXene with *n-*butylamine as the first step in the reaction mechanism (Scheme 4).

**Scheme 4:**
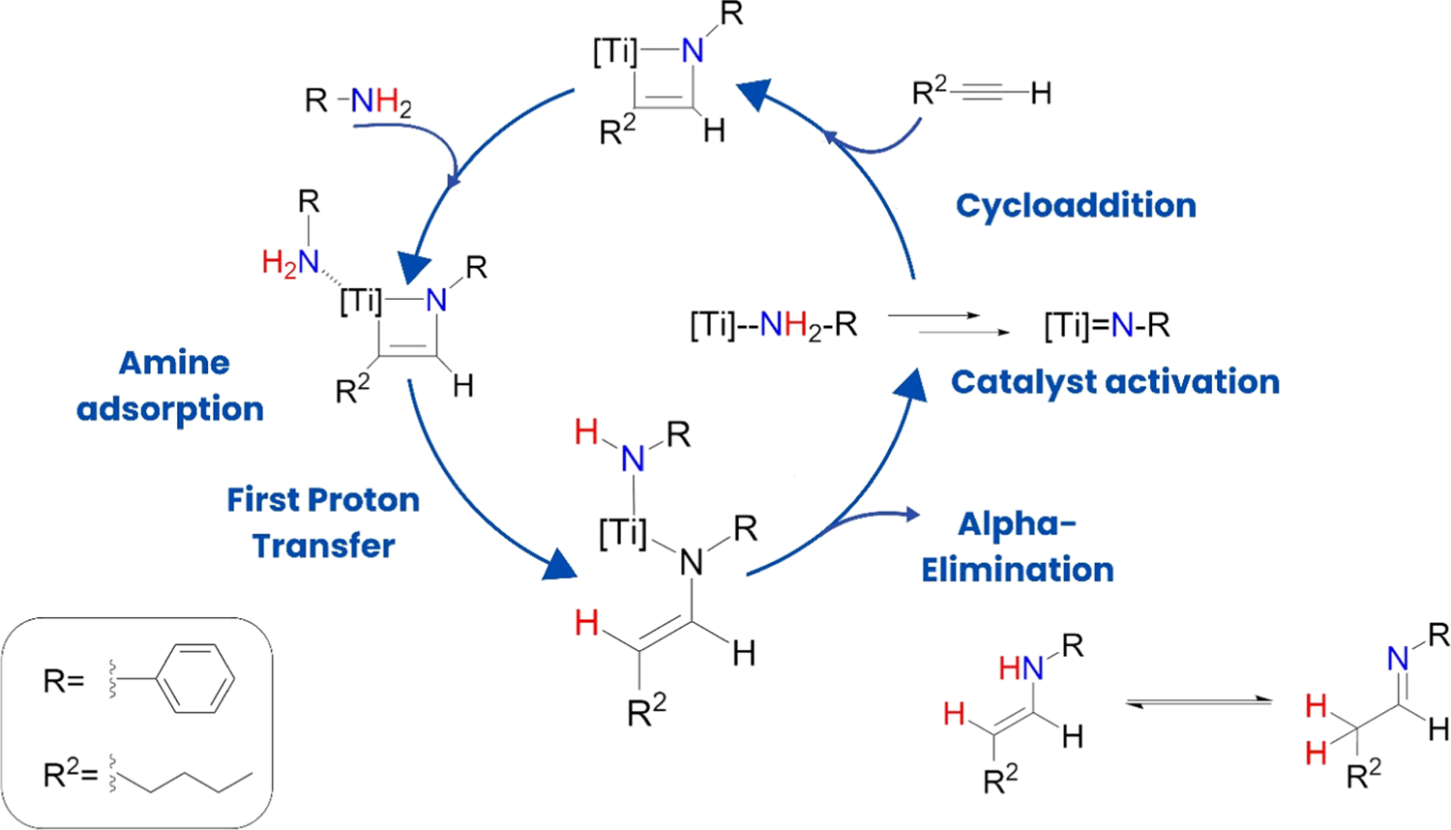
Proposed Hydroamination Reaction Mechanism on the Ti_3_C_2_ Catalyst

The interaction of *n*-butylamine with Ti_3_C_2_ was also supported by solid-state ^13^C NMR spectroscopy by observing a change in the chemical shift of the C-1 carbon from 41.58 ppm for an excessive amount of *n-*butylamine adsorbed on Ti_3_C_2_ in comparison with the 40.34 ppm value measured when only 50 μL of this amine was added to 100 mg of Ti_3_C_2_ (Figure S10). No similar ^13^C NMR spectroscopic shifts were observed for 1-hexyne incorporation into Ti_3_C_2_, indicating that the reaction most likely started with the interaction of the amine with the Ti_3_C_2_ surface.

Based on the Raman and ^13^C NMR data, a mechanism compatible with that previously reported in the literature^93,96^ is proposed in Scheme 4.

To determine the nature of the active sites, pyridine adsorption/desorption measurements monitored by IR spectroscopy were carried out. This quantitative technique distinguishes between the interaction of pyridine with Brönsted and Lewis acid sites by monitoring specific IR vibration bands at 1550 and 1450 cm^–1^, respectively.^97,98^ Furthermore, by measuring the changes in the band intensity upon the desorption temperature in the range from ambient to 400 °C, the acid sites can be ranked as weak, medium, or strong acidity. To the best of our knowledge, such acidity titrations have not yet been reported for MXenes.

In the present case, it was observed that according to pyridine titration, the sample contained both Brönsted and Lewis sites. Figure 8 summarizes these pyridine adsorption–desorption measurements by IR spectroscopy. The strength of these sites, particularly Lewis sites, is medium since the pyridine band intensity diminishes in the range between 200 and 300 °C. Surprisingly, the density of strong Lewis sites undergoes an abrupt increase from 300 °C, in which the population is negligible, to 400 °C, in which strong Lewis acid sites are present. Although the behavior of pyridine adsorption/desorption measurements deserves an in-depth study, the appearance of acid sites between 300 and 400 °C could be attributed to the removal of some surface functional groups in Ti_3_C_2_, generating new acid sites that were absent in the fresh sample. Previous studies on the thermal removal of surface F groups on Ti_3_C_2_ materials obtained by F-etching of Ti_3_AlC_2_ would be in agreement with this proposal.^94^

**Figure 8:**
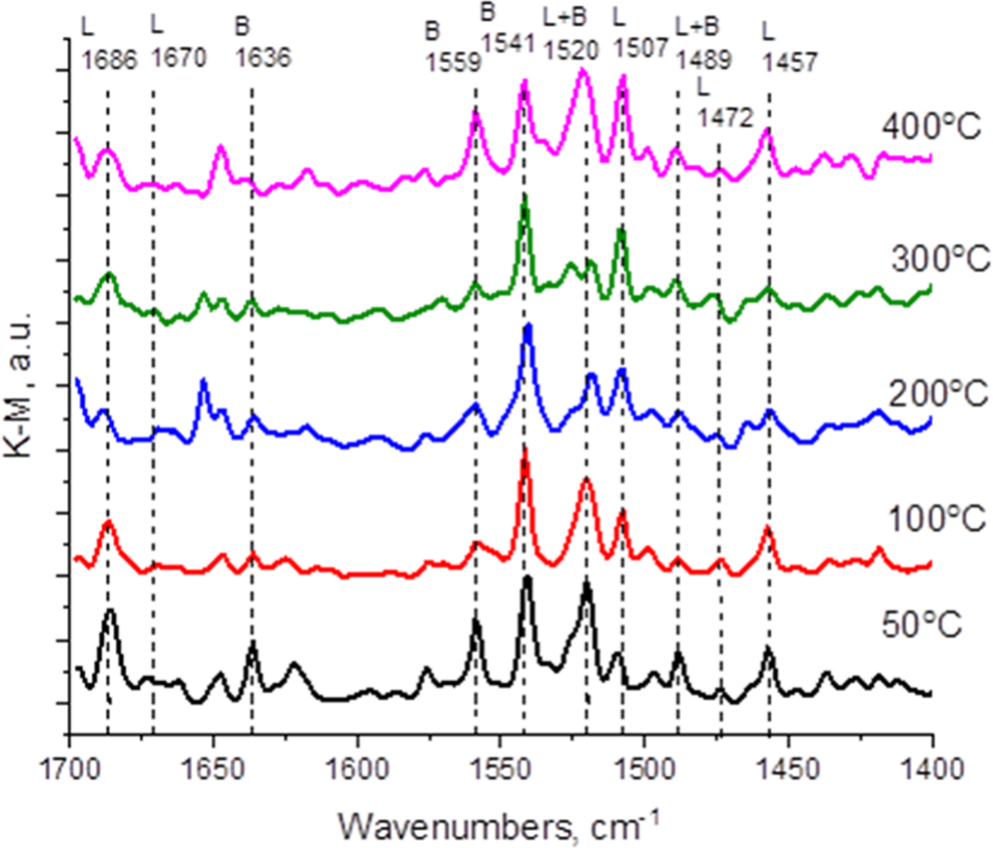
Informative region of the Fourier transform infrared (FT-IR) spectra of Ti_3_C_2_ upon room-temperature adsorption of pyridine and subsequent desorption by thermal treatment at the indicated temperature under dynamic vacuum. The characteristic bands of the Brönsted and Lewis acid sites are labeled. Note the increase in band intensity of the Lewis sites from 300 to 400 °C.

Information on pyridine titration and particularly on the observation of increased pyridine adsorption from 300 to 400 °C suggests that the active sites are exposed Ti atoms lacking surface termination located at the defects and edges of the Ti_3_C_2_ sheets. In this regard, we notice that the lateral size of our sample (80 nm dimension) is considerably smaller than that of other Ti_3_C_2_ samples that have not been subjected to extended ultrasound treatment, which are typically in the micron range.^99^ However, in the present case for catalytic applications, extended exfoliation of the sample achieved by sonication (average thickness of 7 nm by AFM measurements) should be beneficial favoring reagent diffusion, defect generation, and site accessibility.

Considering the proposed nature of the active sites, a preliminary attempt to increase their density by thermal annealing was carried out. It is reported in the literature that the thermal annealing of Ti_3_C_2_ obtained by F-etching can remove some F and OH groups depending on the temperature.^100^ Therefore, this treatment could open further under-coordinated Ti atoms. However, the resulting annealed Ti_3_C_2_ sample at 400 °C under vacuum was devoid of any catalytic activity. It is proposed that the modification of the surface group composition also results in an undesirable increase of the acid strength of Ti atoms by modification of the work function of the material. This proposal is in agreement with the previously reported pyridine adsorption/desorption data, showing the generation of strong Lewis acid sites that would not be required for hydroamination catalysis.

### DFT Calculations

Besides in situ Raman spectroscopy, DFT calculations of the models were performed to determine possible catalytic sites and reasonable pathways. The field of MXenes as electrocatalysts has been dominated by DFT calculations that have provided useful insights into the reaction mechanism and have been used to rationalize the observed results. In the present case, it is important to propose the most likely structure of the catalytic sites.

Our starting point is to consider that a mechanism analogous to that in Schemes 3 and 4 can also be applied to Ti_3_C_2_ as a catalyst with the necessary modifications. These modifications should take into account the constraints imposed by the 2D MXene structure and the presence of Ti neighbors around a given site. In this computational study, we will refer to the hydroamination of 1-hexyne with aniline.

(a) Computational Model: To save computational time, it is common practice in the field of MXene calculations to use Ti_2_C models, even for Ti_3_C_2_.^34,101^ This seems to be reasonable since, as shown earlier, Ti_2_C is also able to catalyze the hydroamination reaction. In the present case, the model for the calculations was extracted from the structure of Ti_2_C MXene. It consists of a cluster of 35 atoms (Ti_11_C_7_F_4_O_7_H_6_) (Figure 9a), which can reproduce the local structure of Ti_3_C_2_ at a reasonable computational cost. This model is also in accordance with the XPS data that shows Ti atoms bonded to C (an intrinsic characteristic of MXenes) as well as bonded to O and F. In addition, XPS O 1s analysis shows a component corresponding to O bonded directly to carbon that has also been considered in the model. (b) First Amine Adsorption: Intermediate A. According to the mechanism depicted in Scheme 4, the first step is the adsorption of aniline onto the MXene cluster. This adsorption would initiate the catalytic cycle, leading to the surface-bound intermediate A, after double deprotonation of aniline (reaction barrier not considered).^93^ The reactive complex formed ([Ti]=N–R, structure A in Scheme 4) would be an unstable species that cannot be isolated during the reaction. The optimized structure is shown in Figure 9b. It can be seen in Figure 9b that the deprotonated aniline is coordinated to three adjacent titanium atoms in the Mxene Ti cluster, differing from the proposed structure ([Ti]=N–R1, coordinated to only one Ti).

**Figure 9:**
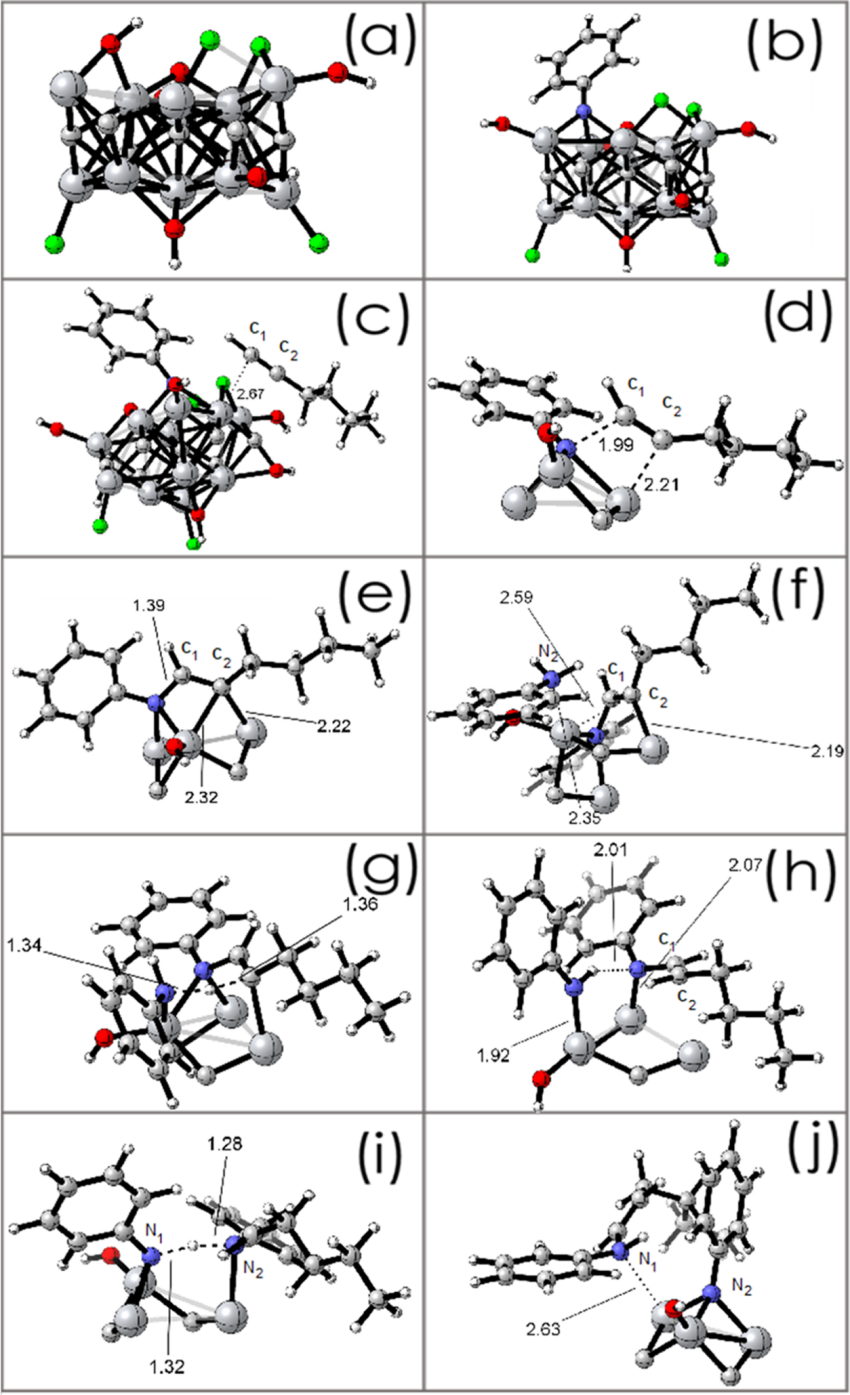
(a) Cluster model of active sites in Ti Mxenes; (b) initial structure of the proposed intermediate “A” indicated in Scheme 4; (c) adsorption of 1-hexyne on the catalytic center (A), with d­(C_1_–Ti) = 2.67 Å and d­(C_2_–Ti) = 2.78 Å; (d) TS of the cycloaddition on the catalytic center, showing d­(N–C_1_) = 1.99 Å and d­(C_2_–Ti) = 2.21 Å; (e) product of the cycloaddition on the catalytic center (structure B in Scheme 4), with d­(N–C_1_) = 1.39 Å, d­(C_2_–Ti_1_) = 2.22 °A, and d­(C2–Ti_2_) = 2.32 Å; (f) adsorption of a second aniline molecule on the catalytic center (B), with d­(N_2_–Ti) = 2.35 Å; (g) TS of the first proton transfer (B-aniline → C), showing d­(N–H) = 1.34 Å and d­(H–C) = 1.36 Å; (h) product of the first proton transfer (C), with d­(C_2_–Ti) = 2.77 Å (not shown); (i) transition state of the second proton transfer (C→ imine A), with d­(N_1_–H) = 1.32 Å and d­(H–N_2_) = 1.28 Å, and (j) product of the second proton transfer (imine A), with d­(N_1_–Ti) = 2.63 Å. For the sake of clarity, structures (d–j) show only a few atoms of the MXene cluster. Red = O, green = F, white = H, gray (big) = Ti, gray (small) = C.

(c) Cycloaddition Reaction, 1-Hexyne + A → B (Scheme 4). The adsorption of 1-hexyne on intermediate A is shown in Figure 9c, with an adsorption enthalpy of −86.5 kJ/mol. The reaction then proceeded to reach the transition state (TS) displayed in Figure 9d, showing an activation energy of 109.9 kJ/mol.

Once the TS barrier has been surpassed, a cycloaddition equivalent product is obtained (Figure 9e, B in Scheme 4), with distances N–Ti 2.15 Å and N–C 1.39 Å, which indicates that the N–C bond has been formed. The distance between C_2_ (the internal carbon of the alkyne) and the closest Ti bonded to N is 2.32 Å. C_2_ is also bonded to an adjacent Ti atom, with a C_2_–Ti_2_ distance of 2.22 Å. This structure is slightly lower in energy than the previous uncycled structure (1-hexyne–A complex) since the overall cyclization energy is exergonic by only 24.9 kJ/mol.

The regiochemistry observed shows that, according to the catalytic complex shown in Figure 9d, 1-hexyne will have a significant steric hindrance for generating the Markovnikov product since the formation of this regioisomer would require the aliphatic chain to move on top of the aromatic ring for the reaction to occur. Therefore, the anti-Markovnikov product (which is the one observed experimentally) is the only one that can approach the titano imine intermediate A with a proper orientation for the reaction to occur, according to the mechanism proposed. Therefore, although other factors, like intercalation between the MXene sheets, Fermi level alteration, and site dispersion, can influence the *regio*selectivity, it seems that the main factor responsible for the anti-Markovnikov selectivity is, as in molecular complexes, the short-range steric hindrance between the reagent substituents.

(d) Second Reaction Intermediate: Adsorption of a Second Ph-NH_2_ Molecule (B + Aniline → B-Aniline). Once the equivalent cycloaddition step takes place, the next step involves the adsorption of a second amine (aniline) to the reaction complex. Subsequent protonolysis of the reaction intermediate (B-aniline) will lead to intermediate C, as shown inScheme [Fig sch4]. The geometry of the adsorbed aniline is shown in Figure 9f, with an enthalpy of −68.7 kJ/mol.

During the coordination of the second aniline, the internal alkyne carbon (C_2_), which was previously coordinated to two Ti atoms, becomes now coordinated to only one, with the other Ti atom being available to coordinate the incoming aniline (wielding N_2_ atom) that adsorbs with an N_2_–Ti distance of 2.35 Å, as shown in Figure 9f. The N_1_–Ti distance is slightly elongated in this process, from 2.15 to 2.19 Å, while the C_2_–Ti distance is shortened from 2.22 (Figure 9f) to 2.19 Å. After adsorption, the alkyne assumes a planar structure, typical of a double bond.

(e) First Proton Transfer (B-Aniline → C). The next step within the catalytic cycle involves proton transfer from the adsorbed aniline to the internal C_2_ carbon, the TS of which is shown in Figure 9g. The TS shows an N–H distance of 1.34 Å, while the H–C distance is 1.36 Å, with an activation energy of 79.3 kJ/mol.

Once proton transfer occurs, the structure of the newly formed intermediate is shown in Figure 9h (C in Scheme 4). The internal alkyne carbon (C_2_) in this intermediate is almost desorbed, as the C_2_–Ti_2_ distance increased to 2.77 Å. The distance between the remaining proton on aniline (N_2_) and the nitrogen of the product (N_1_) is now 2.01 Å.

(f) Second Proton Transfer (C → Imine A). Once the intermediate shown in Figure 9h has been reached, the last step of the reaction mechanism, ignoring the desorption of the formed product, comprises the second proton transfer from aniline to give the adsorbed product (imine A). The structure of the corresponding TS is shown in Figure 9i. As can be seen, in this TS, the N_1_–H distance is 1.28 Å, while the N_2_–C distance is 1.32 Å. The energy barrier is the lowest of the whole mechanism, at 39.4 kJ/mol. This can be due to the greater coordination of the fully deprotonated aniline on the MXene structure, as the N atom is coordinated to two Ti atoms during proton transfer. Moreover, the final product yields deprotonated aniline coordinated to three Ti atoms, resulting in greater stability. The formation of this product is highly exergonic with 190.6 kJ/mol.

Once this last energy barrier is crossed, the structure of the final product (before desorption) is as shown in Figure 9j, where the catalyst regeneration needs only to break the N–Ti bond between MXene and the product.

(g) Global Energy Profile. Considering all reaction steps indicated in Scheme 4, the global energy profile of the aniline hydroamination of 1-hexyne is shown in Figure 10.

**Figure 10:**
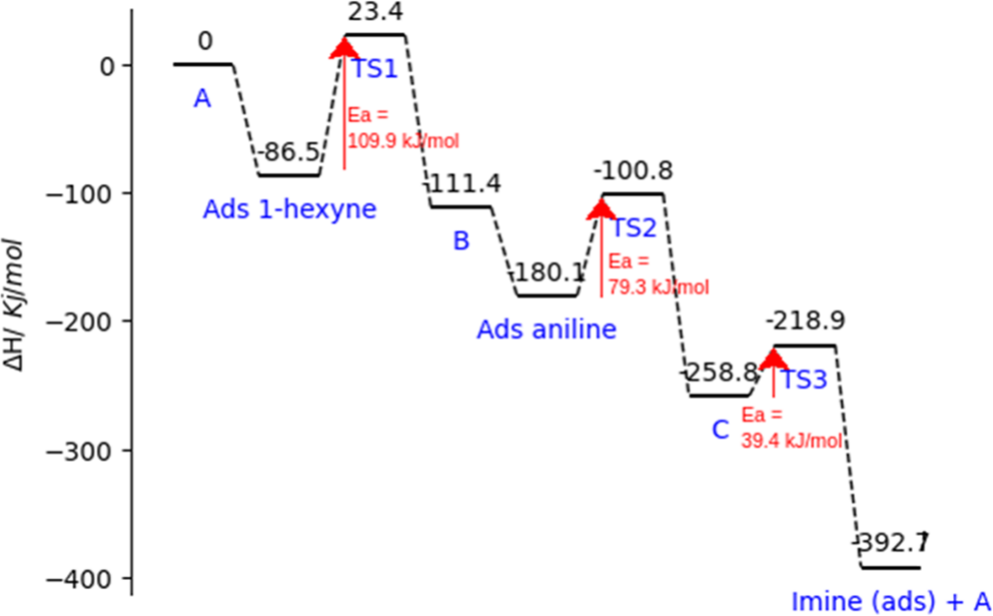
Calculated energy profiles of all reaction intermediates from catalyst A in Scheme 4 to the imine product. Activation barriers corresponding to cycloaddition and proton transfer are indicated by red arrows. Ea = activation energy.

It can be seen that the first TS, corresponding to cycloaddition, has an activation energy of 109.9 kJ/mol, which is the highest of the whole energy profile and, thus, the limiting kinetic step. The first proton transfer has a lower activation energy of about 79.3 kJ/mol. Although we could expect a high barrier for aniline deprotonation, this low barrier was due to the high affinity of the C_2_ carbon for the incipient proton. This is due to the lability of the C–Ti bond (C_2_–Ti in Figure 9d), allowing for the ready formation of C–H. The second proton transfer presents a considerably lower activation energy, 39.4 kJ/mol, probably due to the greater coordination of the incoming aniline yielded during the second proton transfer process. In this step, aniline begins with a −1 formal charge, which is increased to −2 once deprotonation occurs. Although it would be expected that this deprotonation step would have a higher activation energy, this generated negative formal charge would be highly compensated by the relatively uncoordinated neighboring Ti atoms on the MXene surface, at which the aniline rests.

The high activation energy for the cycloaddition could be due to the high steric factors that complicate the access of the active deprotonated aniline site.

This activation energy for the anti-Markovnikov product is expected to be higher for the direct Markovnikov product, as explained above.

## Conclusions

Structural defects are key in heterogeneous catalysis, providing coordinatively unsaturated positions around Lewis acids or basic sites that can interact with substrates. The harsh conditions required for the formation of MXenes from the MAX phase and the large surface area exposed to the ambient environment generate defects, consisting of vacancies of surface functional groups, M or X elements, and atoms at peripheral positions.^102^ Particularly, in the case of the Ti_3_C_2_ sample under study, XPS shows that fluorine, hydroxyl, and oxo groups, as well as vacancies, are present on the surface of the material as terminal groups. These defects are apparently very suitable to promote the regioselective anti-Markovnikov hydroamination of alkynes by aliphatic and aromatic amines, a demanding reaction due to the neutralization of Lewis acid sites by the basic amine, making the general mechanism of electrophilic addition of C–C multiple bonds catalyzed by acids inefficient.^73^ In the case of Ti_3_C_2_, hydroamination reactions occur with very high TOF values of 350 h^–1^, which is significantly higher than that of benchmark catalysts. Ti atoms seem to have an adequate acid strength due to the electron density provided by the negative carbide ions connected to them, making Ti_3_C_2_ an excellent catalyst for the hydroamination of aliphatic and aromatic amines.^50,103^ The present results open the way for further optimization of the catalytic activity by adequate selection of the surface terminal groups and the general use of 2D MXenes as heterogeneous catalysts for other organic reactions. In fact, the interesting observation of oxidative homocoupling of aromatic amines and terminal alkynes supports this claim.

## Supplementary Material



## Notes

MS data of the reaction products; reusability data; hot filtration test data; conversion, selectivity, and TOF of hydroamination of 1-hexyne by aniline, 3,5-dimethyl-, 2,6-dimethyl, and 4-ethylaniline and the corresponding homocoupling products; Raman spectra upon sequential adsorption of aniline and alkynes on Ti_3_C_2_ (PDF)
