# Supplementary material for: MXenes as Heterogeneous Thermal Catalysts: Regioselective Anti-Markovnikov Hydroamination of Terminal Alkynes with 102 h–1 Turnover Frequencies
Source: J Am Chem Soc. 2025 Jan 21;147(4):3315–32. doi: 10.1021/jacs.4c13481 (PMC12123628; doi:10.1021/jacs.4c13481)

Supporting information

MXenes as heterogeneous thermal catalysts. Regioselective anti-Markonikov hydroamination of terminal alkynes with 10^2 h^{-1} turnover frequencies

Rubén Ramírez Grau[1], Pablo Garcia-Aznar[1], German Sastre[1], Sara Goberna-Ferrón,[1] Octavian Pavel,[2] Alina Tirsoaga[2], Bogdan Cojocaru[2], Dana Georgeta Popescu[3], Vasile I. Parvulescu*[2], Ana Primo*[1], Hermenegildo García*[1]

[1] Instituto Universitario de Tecnología Química, Universitat Politècnica de València-Consejo Superior de Investigaciones Científicas, Universitat Politècnica de València, Av. De los Naranjos s/n, 46022 Valencia, Spain

[2] Department of Organic Chemistry, Biochemistry and Catalysis, University of Bucharest, B-dul Regina Elisabeta 4-12, Bucharest 030016, Romania

[3] National Institute of Materials Physics, 405A Atomistilor Str., Magurele 077125, Ilfov, Romania

MS data of reaction products.

N-phenylhexanimines m/z: 32.02(20.53), 39.07(4.32), 42.08(7.62), 51.05(16.09), 77.06(69.52), 78.07(5.97), 91.06(8.97), 92.08(23.76), 93.08(4.84), 104.06(11.98), 117.08(7.63), 118.07(59.08), 119.08(6.63), 130.06(6.01), 132.08(100), 133.1(76.17), 134.11(7.19), 146.09(15.13), 160.11(7.59), 175.12(10.79), dodeca-5,7-diyne (m/z 32.04(37.53), 39.07(10.37), 41.07(20.42), 51.06(12.57), 55.07(9.15), 63.04(12.57), 65.07(14.78), 67.09(9.9), 77.07(35.32), 78.08(31.19), 79.08(31.34), 91.06(100), 92.08(24.41), 93.09(13.05), 103.07(10.4), 105.07(62.49), 106.09(9.97), 117.08(10.08), 119.09(15.78), 162.14(34.59), phenyldiazene (m/z: 32.03(85.29), 39.13(5.29),

40.03(6.79), 44.03(7.45), 50.07(5.51), 51.04(22.24), 65.05(5.01), 66.1(5.6), 77.05(100), 78.1(8.28), 79.08(5.82), 91.09(8.96), 92.08(6.38), 93.08(13.14), 105.07(22.96), 121.04(4.97), 152.06(11.43), 153.1(6.75), 182.07(43.66));

N-(3,5-dimethylphenyl)hexanimines m/z: 39.07(12.47), 40.04(9.81), 51.06(10.3), 65.12(14.22), 77.06(44.37), 78.11(13.47), 79.07(39.08), 91.09(32.97), 103.07(18.1), 105.1(42.31), 106.1(59.38), 120.12(54.22), 121.11(50.03), 134.12(27.71), 146.12(36.95), 149.1(70.5), 160.12(100), 161.14(57.29), 174.14(14.51), 203.19(12.25);

N-(3,5-dimethylphenyldiazenes (m/z: 39.08(3.68), 40.02(3.5), 51.04(3.11), 65.06(3.39), 77.06(19.99), 78.1(5.39), 79.07(18.48), 91.08(5.01), 103.07(11.64), 104.05(3.44), 105.08(100), 106.1(10.28), 121.11(3.14), 133.08(7.45), 165.1(5.6), 179.12(4.27), 180.12(5.41), 195.14(3.46), 238.18(30.22));

*N-(*2,6-dimethylphenyl)hexanimines (m/z: 77.07(6.66), 79.09(6.65), 91.05(4.16), 103.08(5.64), 105.1(11.98), 120.09(3.13), 131.1(11.18), 132.1(6.93), 144.08(5.09), 146(25.3), 147(31.21), 149.1(68.21), 160.11(100), 161.15(48.31), 174.09(4.39), 188.13(6.03), 203.12(12.34)); 2,6-dimethylphenyldiazenes (m/z relative: 39.08(13.57), 40.02(5.4), 51.04(2.1), 65.06(3.51), 77.06(12.89), 78.1(3.27), 79.07(5.32), 91.08(6.01), 102.06(4.2), 103.07(3.54), 105.08(100), 106.1(5.35), 121.11(2.38), 133.08(4.32), 165.1(4.58), 179.12(5.38), 180.12(6.53), 195.14(7.58), 238.18(10.12));

*N-(*4-ethylphenyl)hexanimines (m/z relative: 77.06(29.97), 78.08(9.61), 79.08(25.02), 90.1(8.43), 91.1(16), 103.09(17.22), 105.11(40.17), 106.1(28.86), 120.12(27.94), 121.13(10.2), 132.11(16.59), 134.08(15.62), 146.12(59.29), 149.1(16.99), 160.14(100), 161.15(92.2), 162.15(11), 174.12(20.89), 188.15(8.07), 203.17(19.45)); 4-ethylphenyldiazene (m/z relative: 39.06(2.81), 40.02(3.93), 44.02(2.96), 51.05(3.67), 65.07(2.82), 77.06(21.97), 78.10(5.53), 79.09(23.51),

89.06(3.9), 90.09(2.39), 91.1(4.08), 103.08(16.15), 105.09(100), 106.12(10.38), 133.09(19.12), 165.07(4.21), 180.11(3.89), 238.15(34.74)).

Table S1. EXAFS data fitting results of Samples.

Sample	Path	CN^a	$R(\text{Å})^b$	$\sigma^2\ (\text{Å}^2)^c$	$\Delta E_0(\text{eV})^d$	R factor
Ti K-edge (S_0^2=0.786)						
Ti foil	Ti -Ti	12*	2.907±0.004	0.0084	5.3	0.0058
TiO2_Anatase	Ti -O	6.0±0.1	1.968±0.018	0.0022	7.05	0.0031
	Ti -O-Ti	4,1±0.2	2.949±0.016	0.0062		
	Ti -O-Ti	4.0±0.3	3.889±0.024	0.0134		
Ti2O3	Ti -O	5.8±0.1	2.064±0.012	0.0056	0.7	0.0086
	Ti -O-Ti	4.2±0.3	2.931±0.019	0.0064		
TiC	Ti -C	6.0±0.1	2.173±0.018	0.0054	-6.7	0.0092
	Ti -C-Ti	12.1±0.3	3.048±0.004	0.0075		
3-Ti3C2-Ti-1	Ti -C/O	4.4±0.5	1.954±0.013	0.0023	-7.0	0.0051
	Ti -C/O	1.5±0.2	2.301±0.012	0.0056		
	Ti -C-Ti	3.6±0.3	3.057±0.019	0.0123		

$^a CN$, coordination number; $^b R$, the distance between absorber and backscatter atoms; $^c\sigma^2$, the Debye Waller factor value; $^d\Delta E_0$, inner potential correction to account for the difference in the inner potential between the sample and the reference compound; R factor indicates the goodness of the fit. $S0^2$ was fixed to 0.914 and 0.786, according to the experimental EXAFS fit of Ti foil by fixing CN as the known crystallographic value. * This value was fixed during EXAFS fitting, based on the known structure of Ti. Fitting conditions: k range：3.0 - 14.0; R range: 1.4-3.5 (3-Ti3C2-Ti-1); fitting space: R space; k-weight = 3. A reasonable range of EXAFS fitting parameters: $0.800 < S_0^2 < 1.000$; $CN > 0$; $\sigma^2 > 0$ Å^2; $|\Delta E_0| < 15$ eV; R factor < 0.02

Table 2. Results of the catalytic activity of Ti_3C_2. Reaction conditions: 150 °C, 2 mL toluene, 24 h, 0.001 moles alkyne, 0.002 moles amine, 5 mg catalyst, autoclave.

Alkyne	Amine	Conversion[a] (%)	Hydroamination selectivity (%)	TON	Remarks
1-hexyne	4-aminophenol	0	--	--	No hydroamination product
	4-nitroaniline	0	--	--	
1-phenylacetylene	2-Methyl-aniline	93	13	25.7	Selectivity towards anti Markovnikov product
	3-Methyl-aniline	93	14	26.8	
4-octyne	3-methylaniline	0	--	--	No hydroamination product
1-phenyl-1-hexyne	3-methylaniline	0	--	--	

[a] Based on alkyne

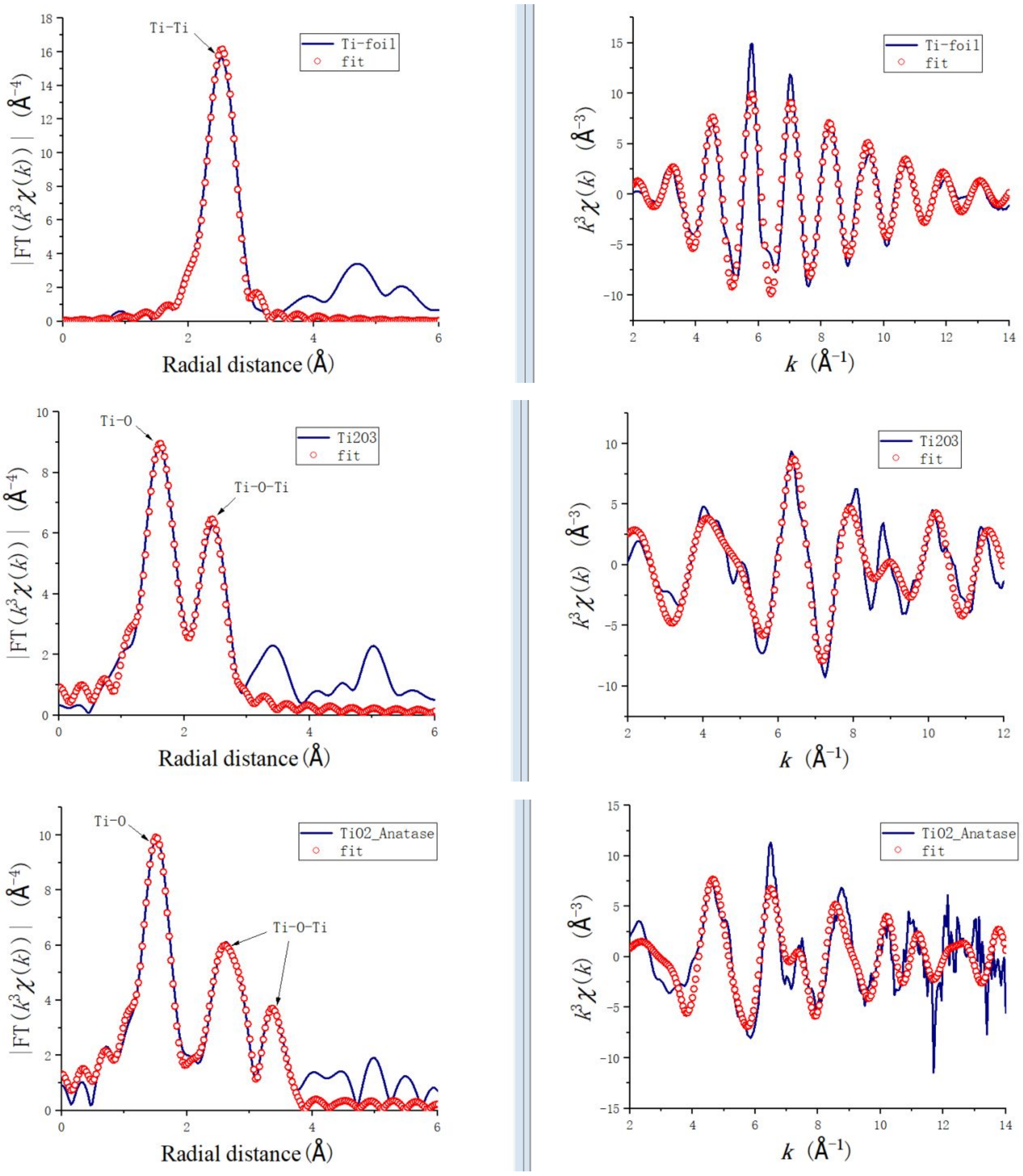

Supporting information 6

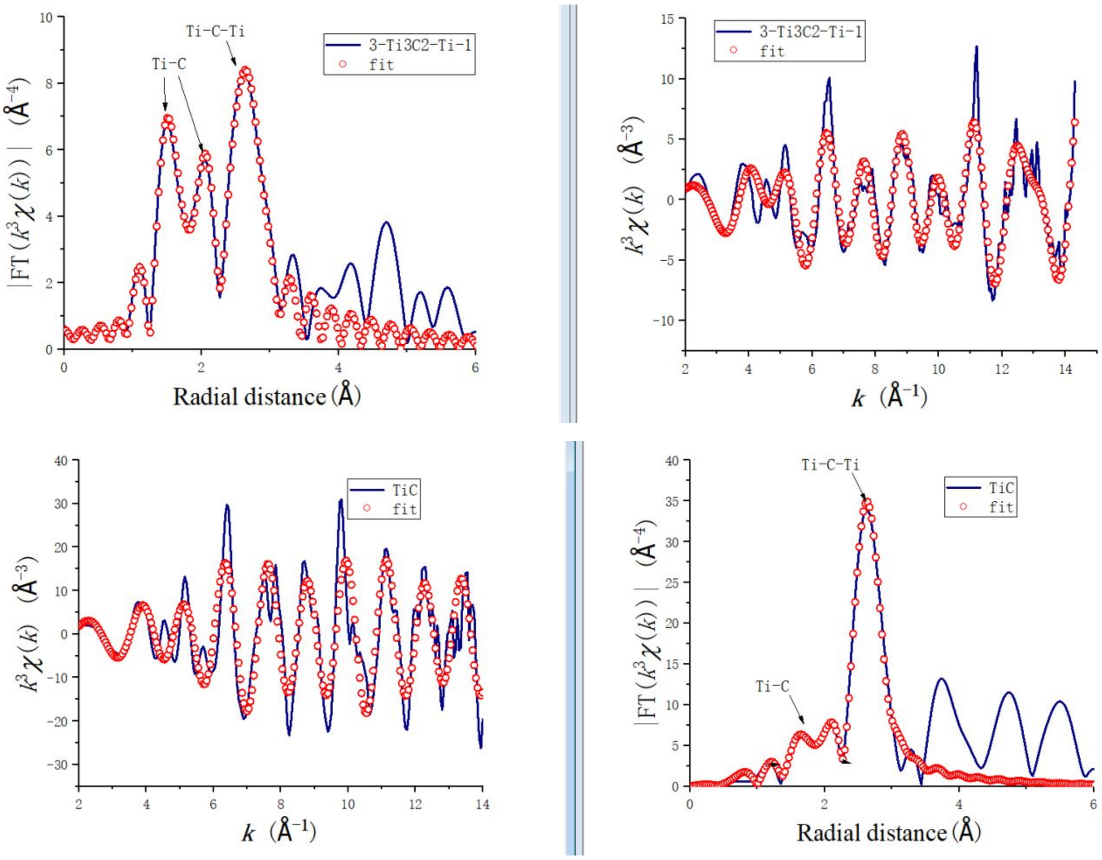

Figure S1. Fitting of the magnitude of the Fourier transform of the k3-weighted EXAFS (data-blue and fit-red) for Sample and Standards and the corresponding EXAFS fitting curves of Sample and Standards at k space.

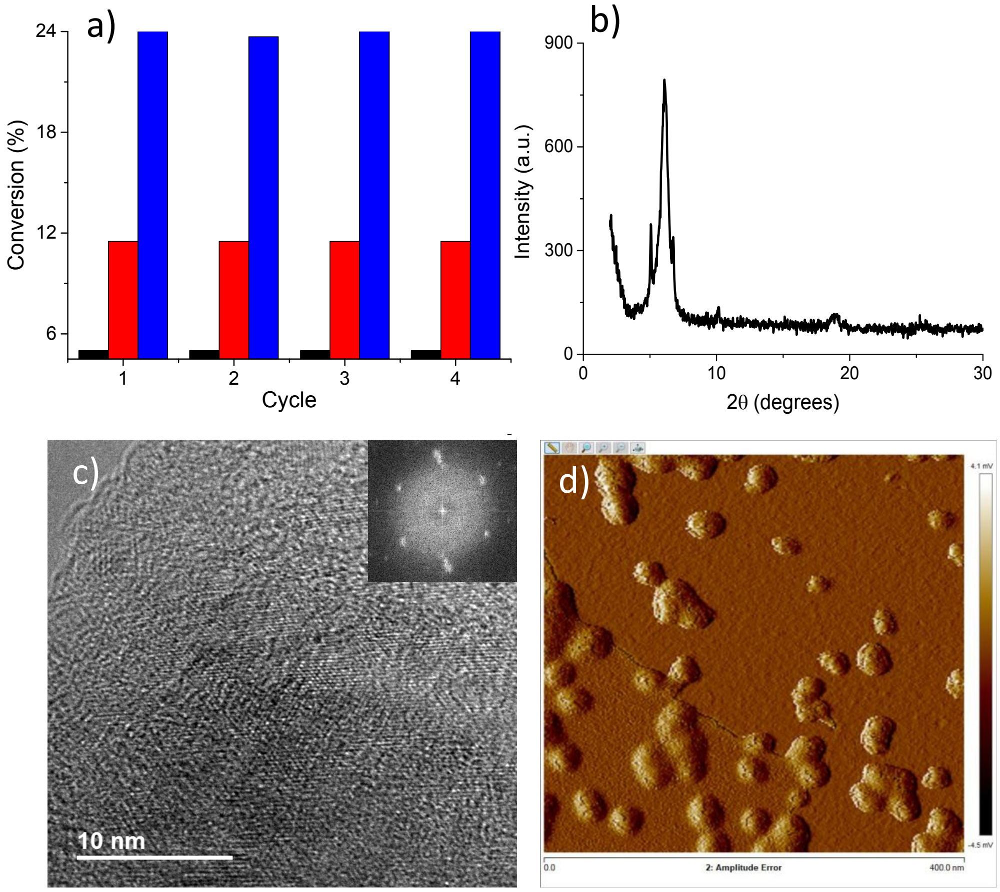

Figure S2. a) Catalytic activity of the same Ti_3C_2 sample in four consecutive reuses for the hydroamination of 1-hexyne by *n*-butylamine under various conditions: black bars: 140 ^oC, 24 h; red bars: 160 ^oC, 24 h; blue bars: 160 ^oC, 48 h. Reaction conditions: 5 mg Ti_3C_2, 1 mmol aniline, 2 mmol n-butylamine, 2 mL toluene. b) XRD (b), TEM (c) and AFM (d) of the four times used Ti_3C_2 sample. The inset of frame c corresponds to the electron diffraction pattern of the image, showing that crystallinity has been maintained.

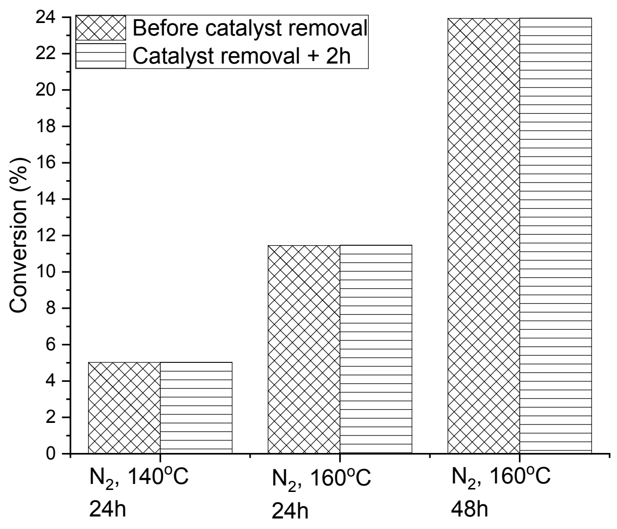

Figure S3. Hot filtration test for the hydroamination of 1-hexyne by *n*-butylamine under various conditions where it can be seen that in all cases the reaction stops upon removal of the Ti_3C_2 catalyst.

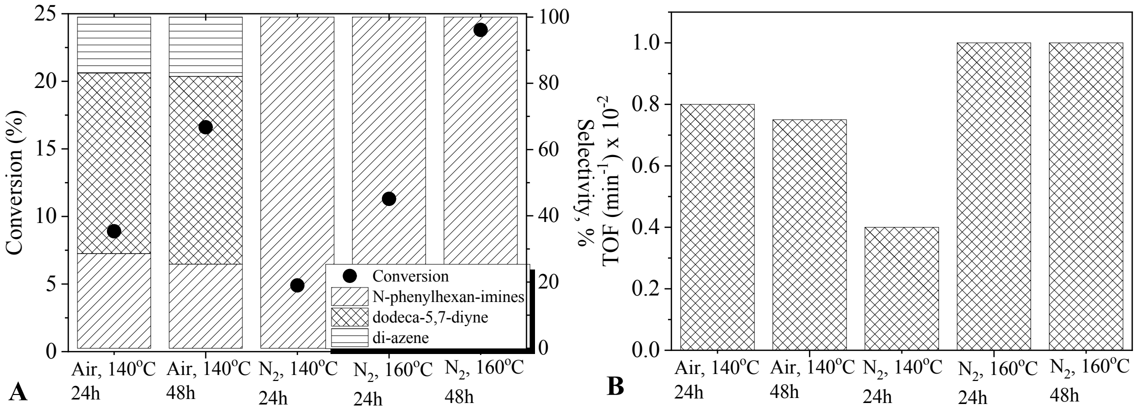

Figure S4. Hydroamination of 1-hexyne with aniline and homocoupling products under various conditions (A) variation of the conversion and selectivity, (B) variation of the TOF. Reaction conditions: 1 mmol 1-hexyne, 2 mmols aniline, toluene as solvent.

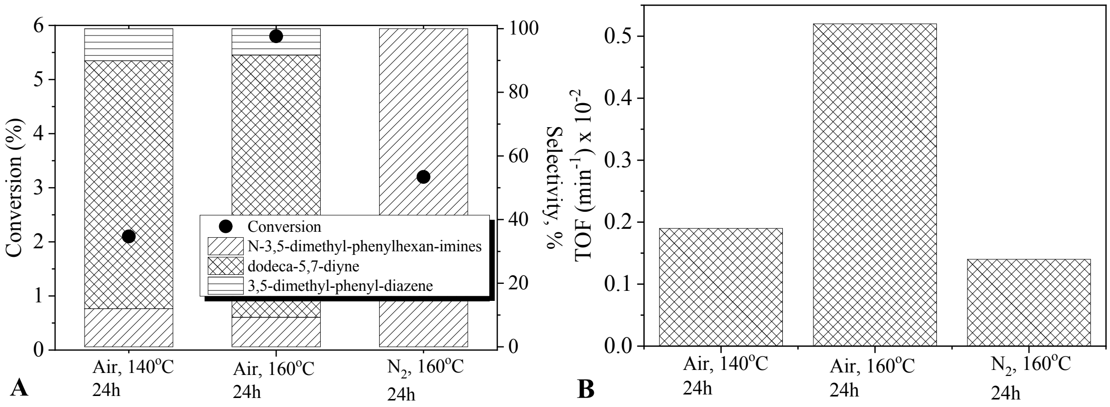

Figure S5. Hydroamination of 1-hexyne with 3,5-dimethylaniline and homocoupling products under various conditions (A) variation of the conversion and selectivity, (B) variation of the TOF. Reaction conditions: 1 mmol 1-hexyne, 2 mmols 3,5-dimethylaniline, toluene as solvent.

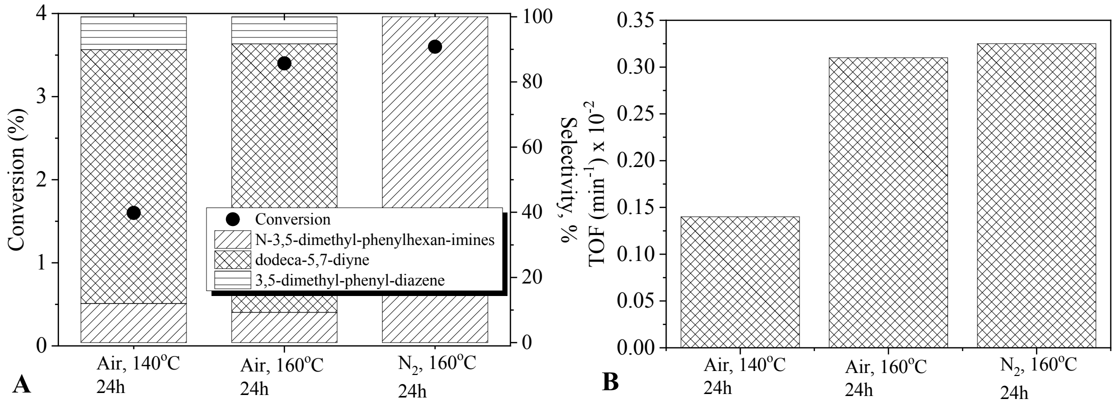

Figure S6. Hydroamination of 1-hexyne with 2,6-dimethylaniline and homocoupling products under various conditions (A) variation of the conversion and selectivity, (B) variation of the TOF. Reaction conditions: 1 mmol 1-hexyne, 2 mmols 2,6-dimethylaniline, toluene as solvent.

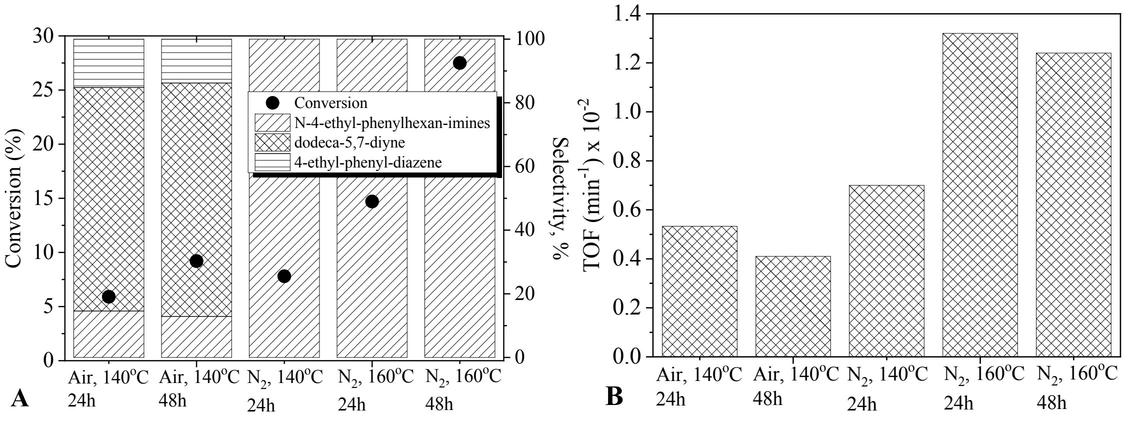

Figure S7. Hydroamination of 1-hexyne with 4-ethylaniline and homocoupling products under various conditions (A) variation of the conversion and selectivity, (B) variation of the TOF. Reaction conditions: 1 mmol 1-hexyne, 2 mmols 4-ethylaniline, toluene as solvent.

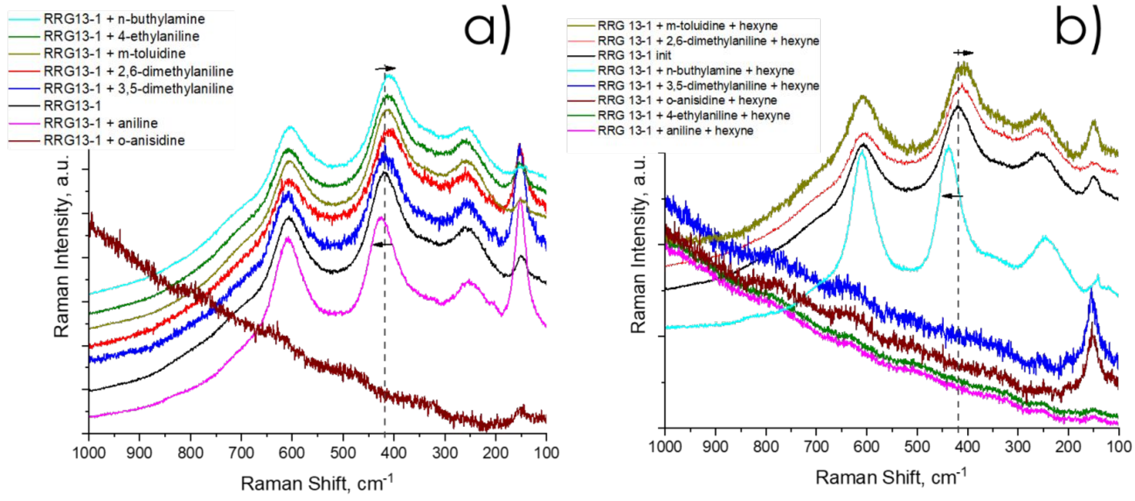

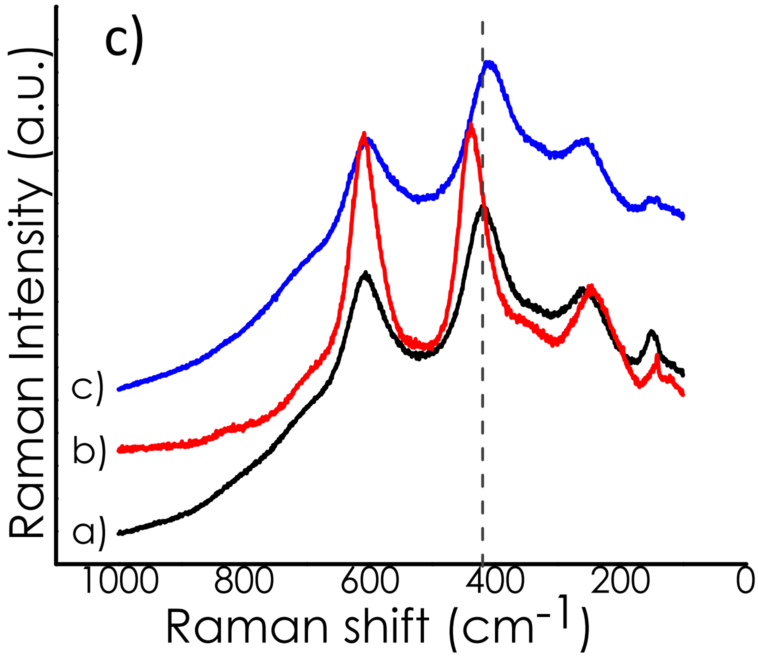

Figure S8. Raman spectra (488 nm excitation) upon adsorbing aromatic amines as indicated in the plot on Ti_3C_2 in toluene at 100 °C for 30 min, followed by toluene washings and drying (a) and subsequent addition of 1-hexyne (b). The vertical dashed line is a guide showing the position of the 420 cm^{-1} band of the fresh Ti_3C_2 sample. Absence (a) or disappearance (b) of the Raman signal in some cases is proposed to be due to fluorescence caused by 488 nm laser excitation. c) Expanded low frequency zone of the Raman spectra (488 nm excitation) of: a) initial Ti_3C_2; b) upon adsorbing n-butylamine on Ti_3C_2 in toluene at 50 °C for 30 min, washing with toluene and drying, and c) subsequent addition of 1-hexyne. The vertical dashed line corresponds to the position of the maximum intensity of the 420 cm^{-1} band of the fresh Ti_3C_2 sample.

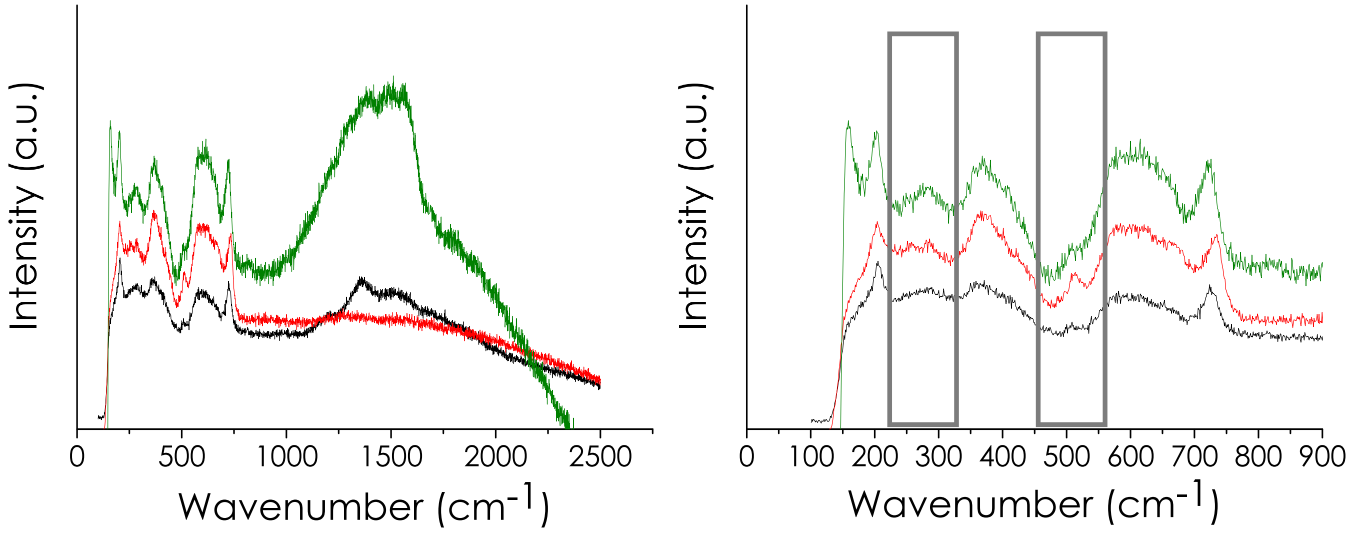

Figure S9. Full Raman spectra (left) and corresponding expanded region of surface functional groups (right) upon 785 nm laser excitation of pristine Ti_3C_2 (black), after absorption of 1-butanamine to pristine Ti_3C_2 (red) and subsequent addition of 1-hexyne (green). The two rectangles of the right plot highlight the differences with the appearance in the red spectrum of three new peaks at 505, 270 and 260 cm^{-1}.

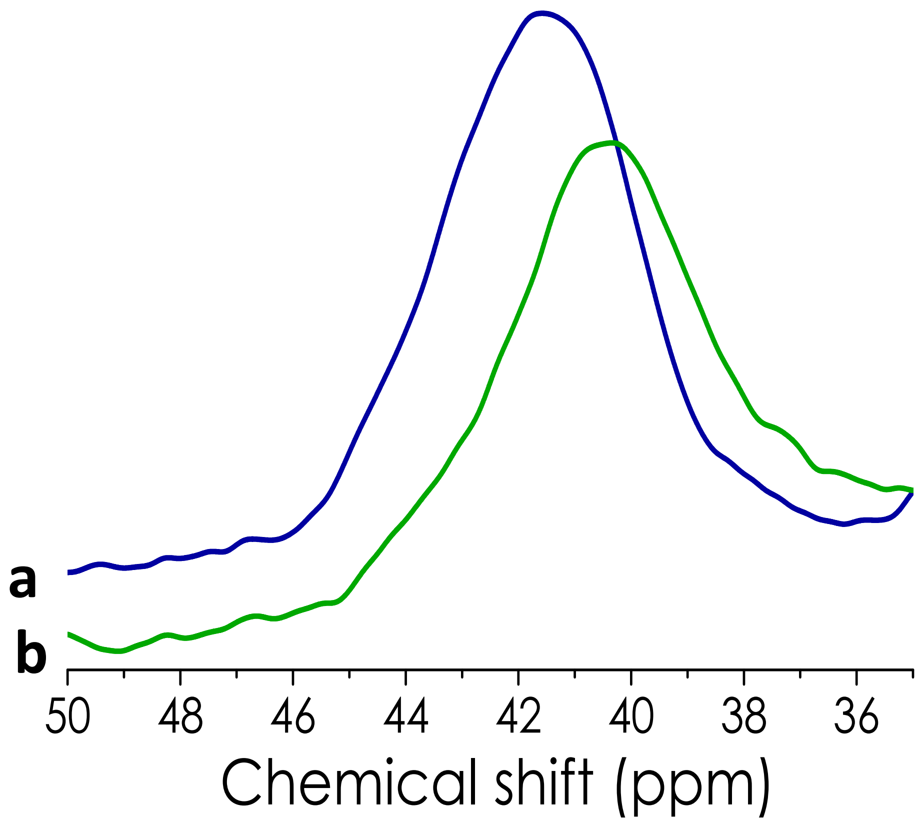

Figure S10. Expansion of the region corresponding to C_1 in 1-butanamine of the solid state ^{13}C-NMR spectra upon adsorbing either 10 ml (plot a in blue, δ 41.58 ppm) or 50 µl (plot b in green δ 40.34 ppm) onto Ti_3C_2 (100 mg).

RAW DATA

Isotherm N_2 adsorption data to determine Ti_3C_2 surface area (BET)

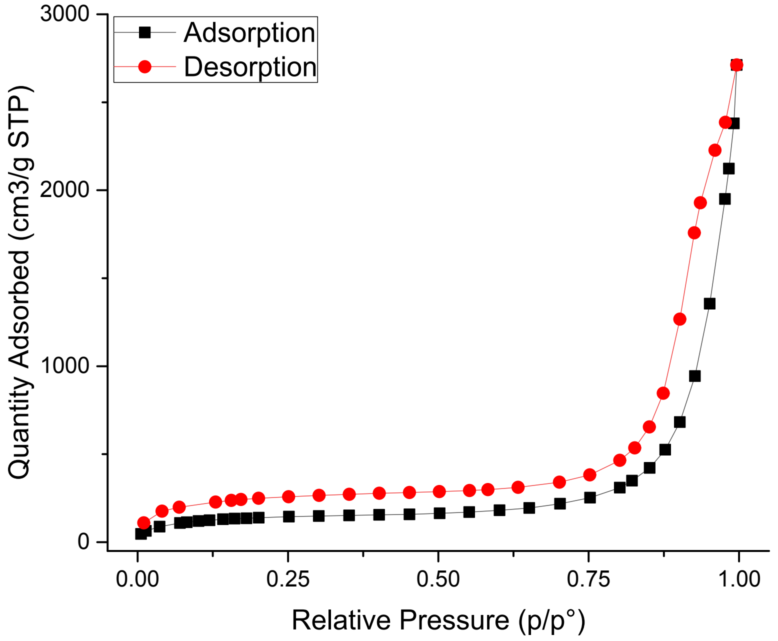

Pore size distribution of Ti_3C_2

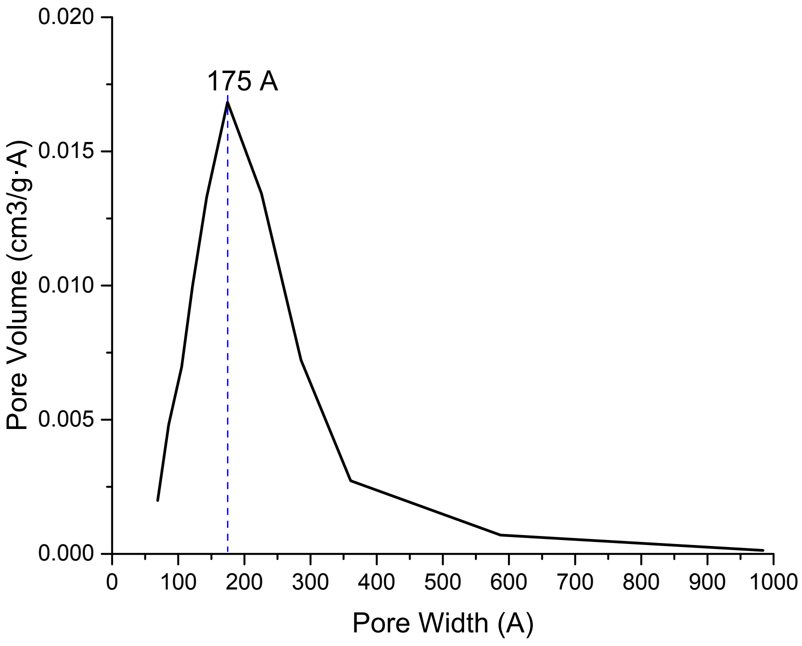

Particle size distribution of Ti_3C_2 suspended in aqueous solution measured by dynamic laser scattering.

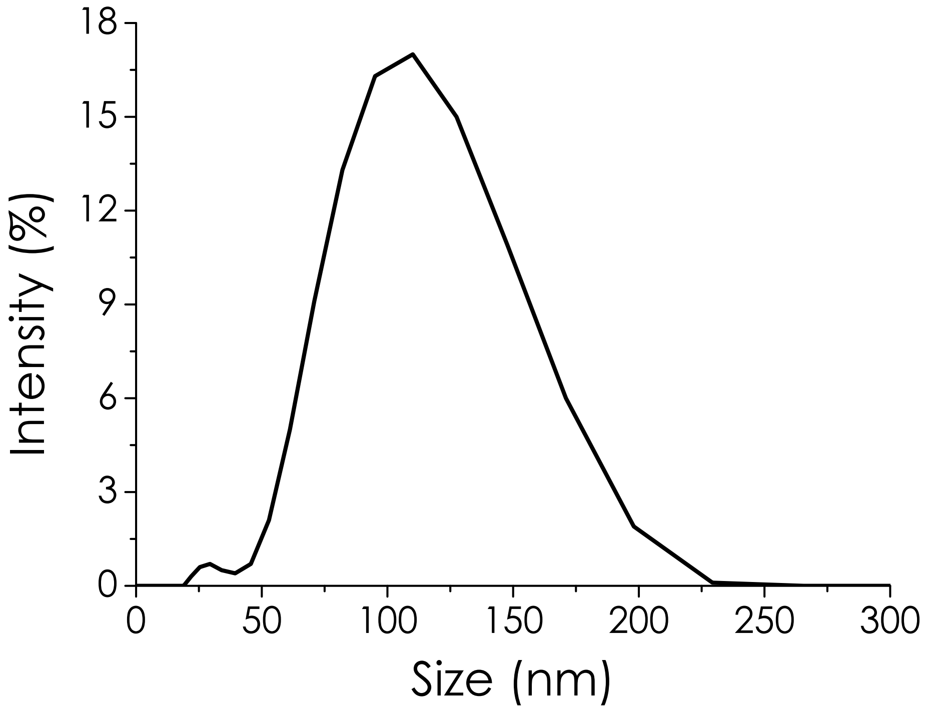

NH_3-TPD spectra of Ti_3C_2

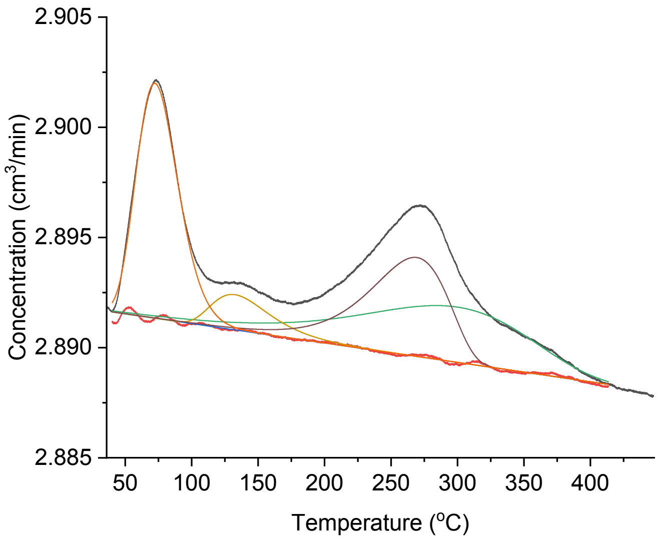

CO_2-TPD spectra of Ti_3C_2

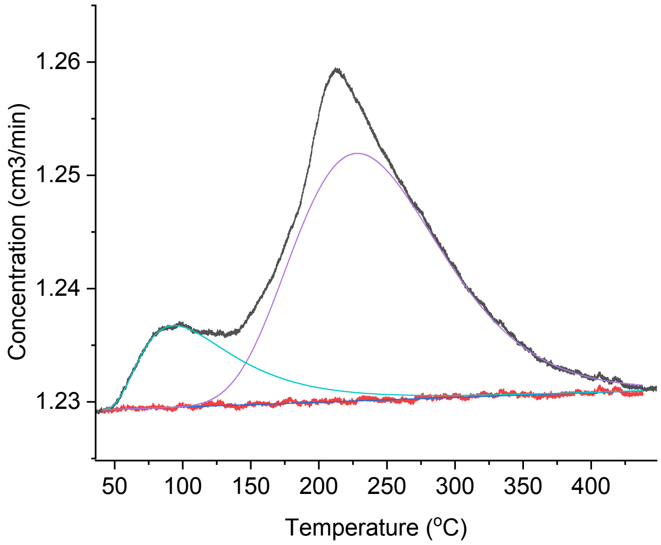

H_2-TPD spectra of Ti_3C_2

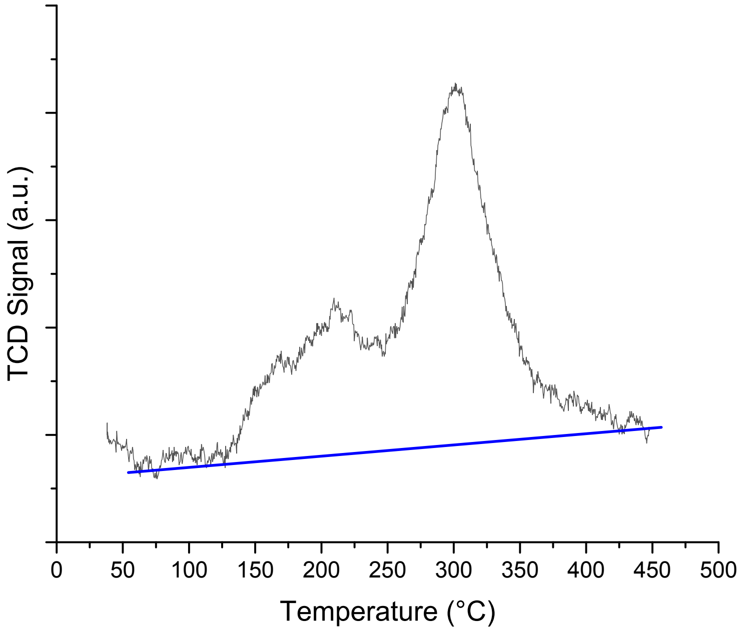

Raman spectra of the Ti_3C_2 catalyst using different excitation wavelengths: 325 nm (red line) 488 nm (green line) and 633 nm (blue line)

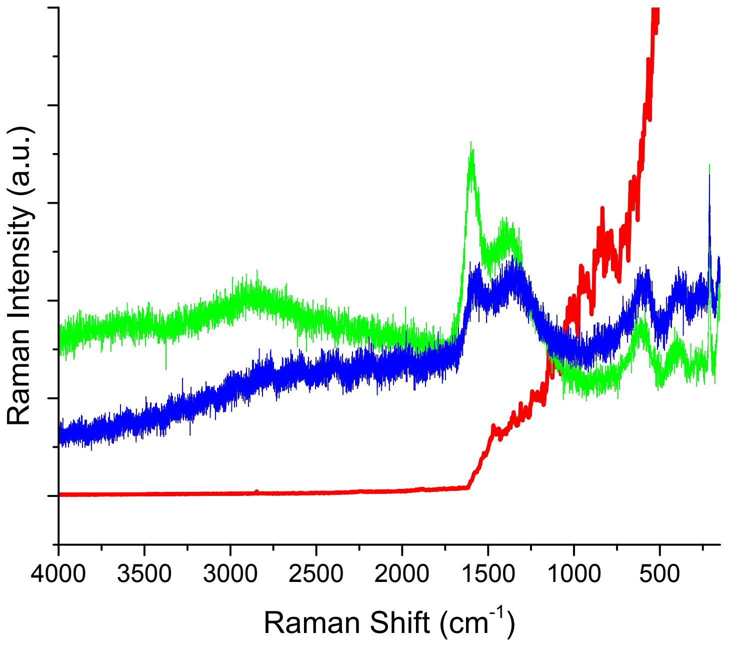

Raman spectra of the Ti_3C_2 before (black line) and after (red line) its use as catalyst.

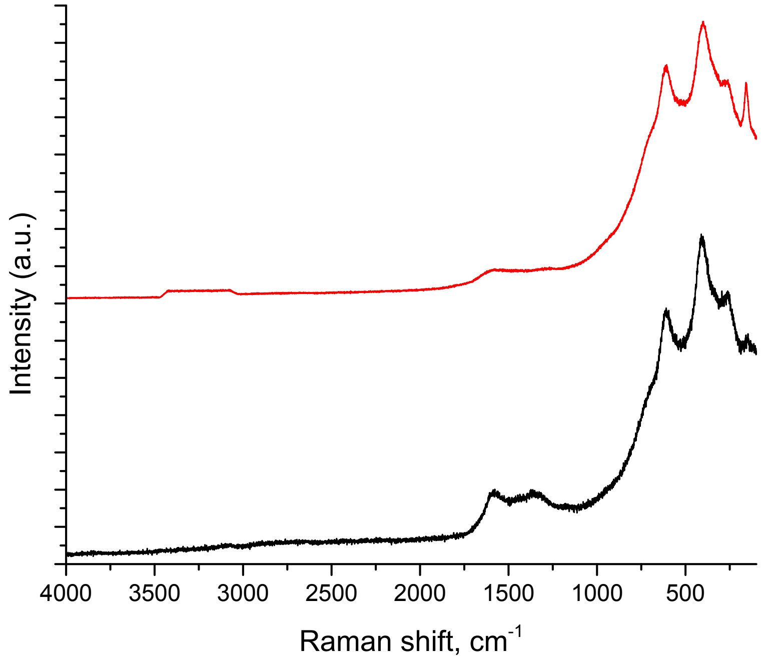

Raman spectra upon adsorbing aromatic amines as indicated in the plot on Ti_3C_2 by 488 nm wavelength exitation

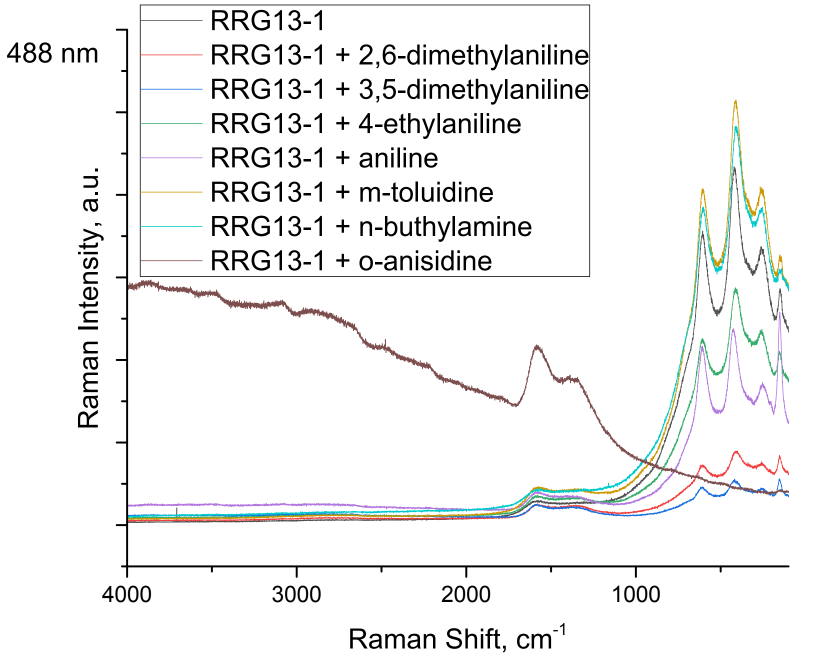

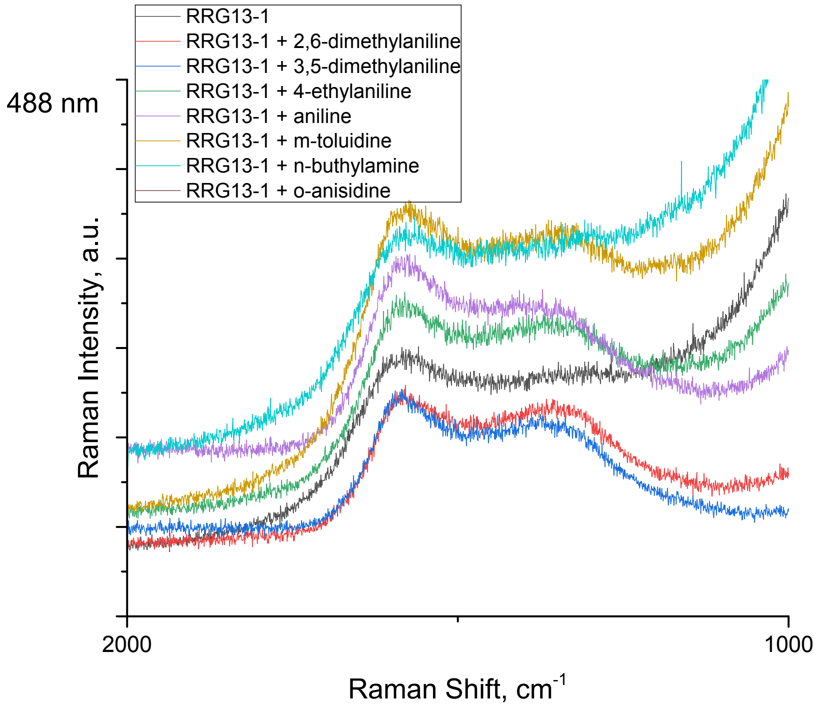

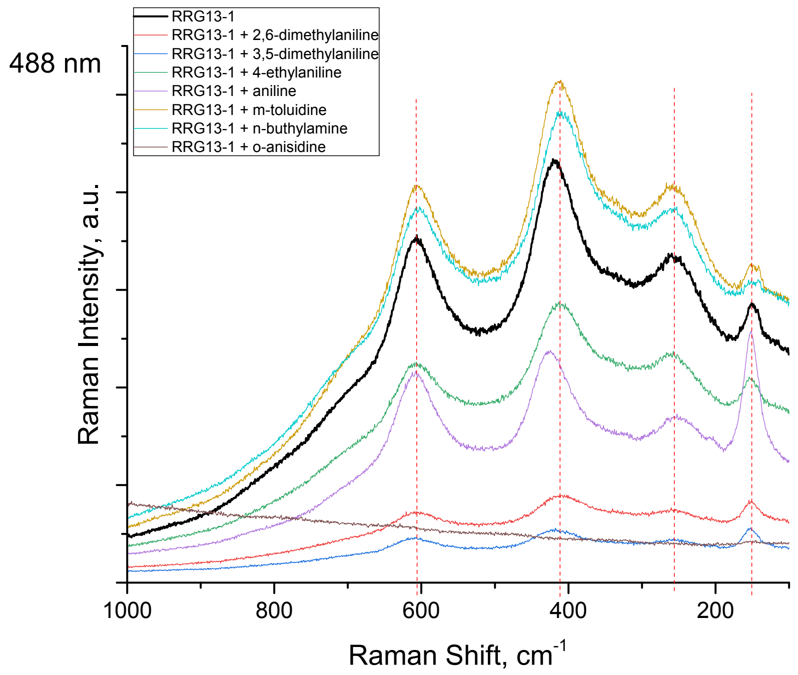

Raman spectra upon adsorbing aromatic amines and addition of 1-hexyne as indicated in the plot on Ti_3C_2 by 488 nm wavelength excitation

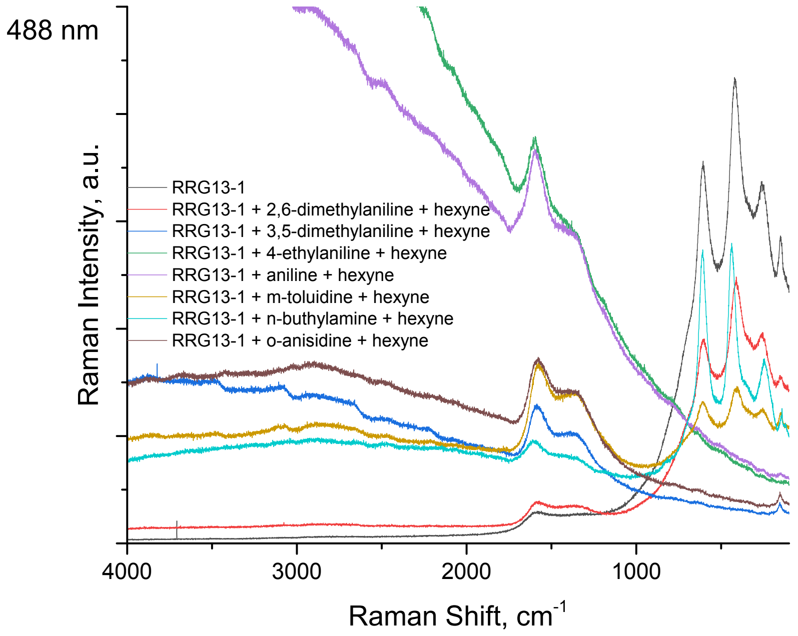

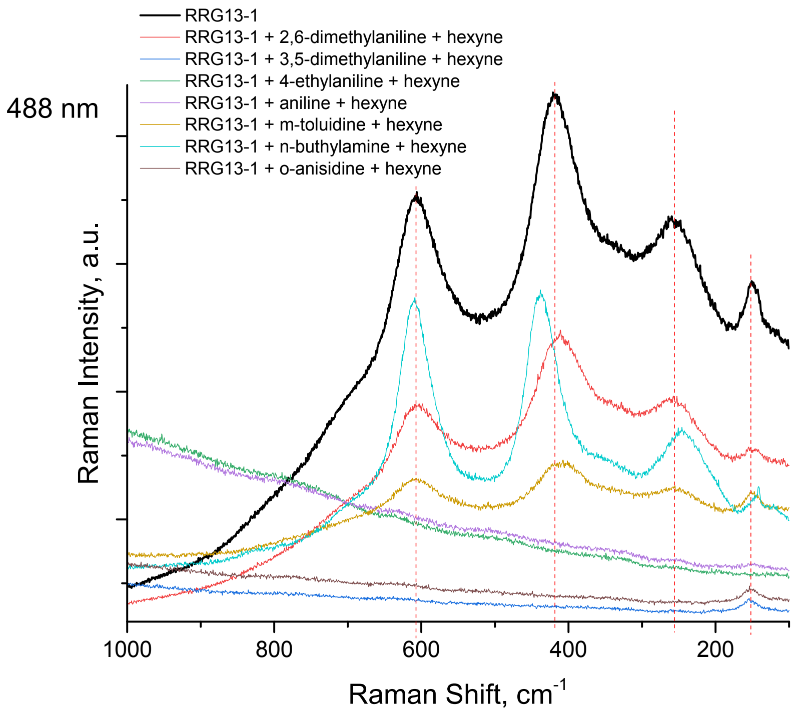
RRG13-1
RRG13-1 + 2,6-dimethylaniline + hexyne
RRG13-1 + 3,5-dimethylaniline + hexyne
RRG13-1 + 4-ethylaniline + hexyne
RRG13-1 + aniline + hexyne
RRG13-1 + m-toluidine + hexyne
RRG13-1 + n-buthylamine + hexyne
RRG13-1 + o-anisidine + hexyne
488 nm
Raman Intensity, a.u.
1000
800
600
400
200
Raman Shift, cm-1

<u>Raman spectra upon adsorbing aromatic amines as indicated in the plot on Ti_3C_2 by 633 nm wavelength excitation</u>

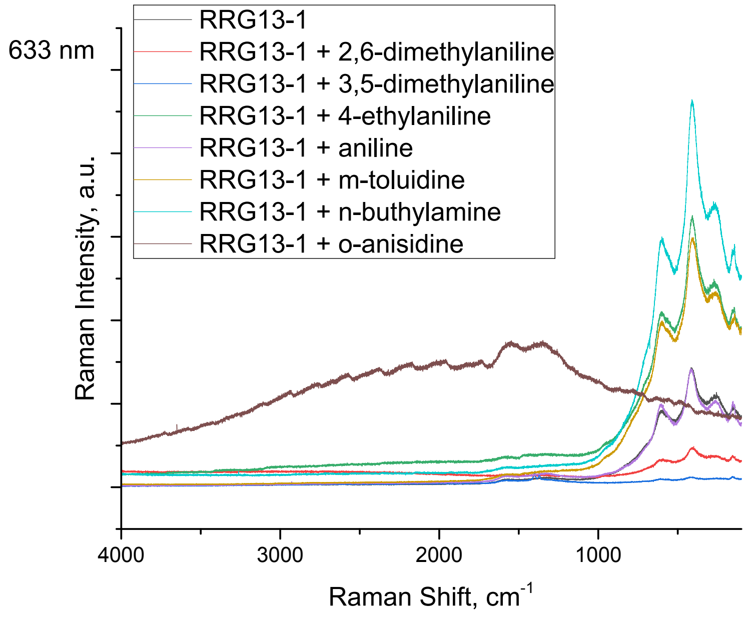

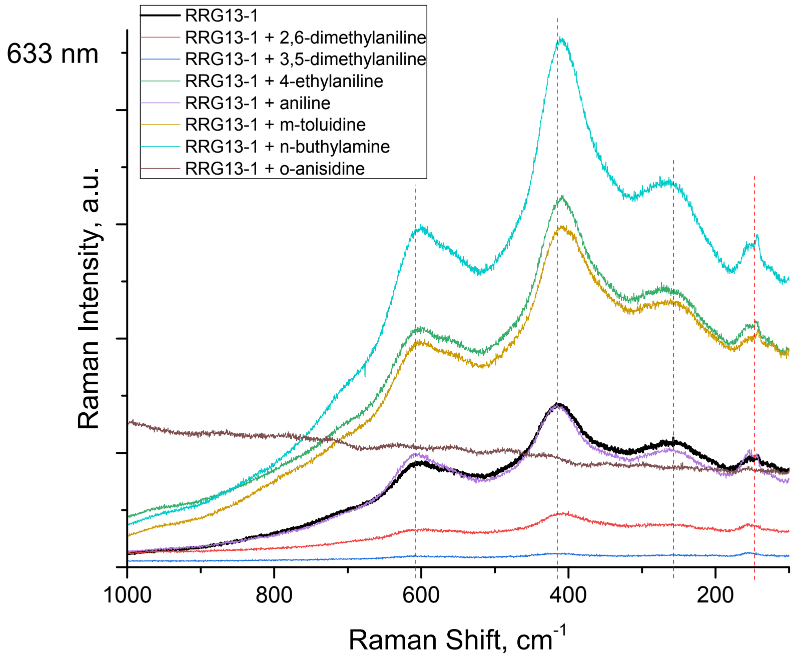

Raman spectra upon adsorbing aromatic amines and addition of 1-hexyne as indicated in the plot on Ti_3C_2 by 633 nm wavelength excitation

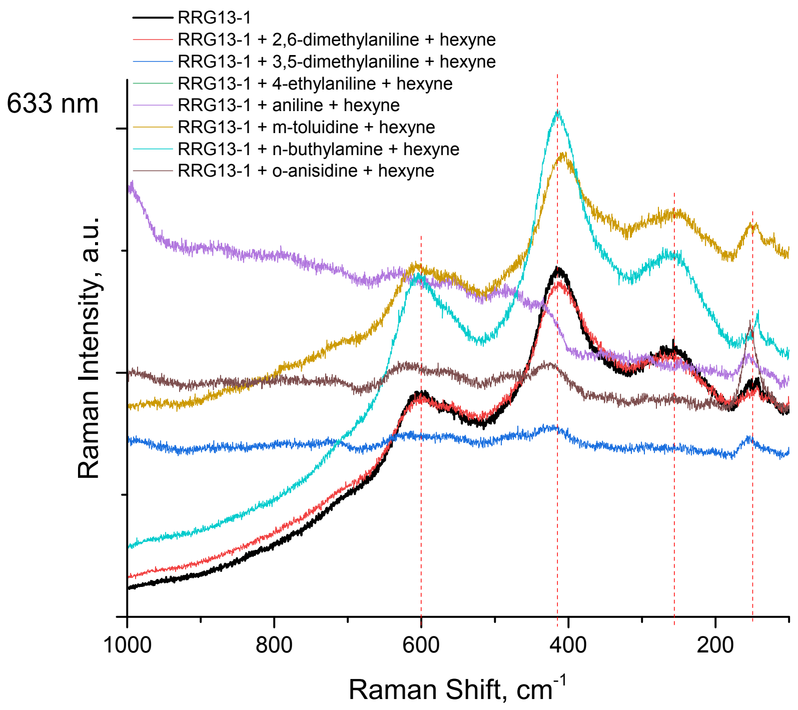

CHROMATOGRAMS

Reaction of phenylacetylene and 2-methylaniline

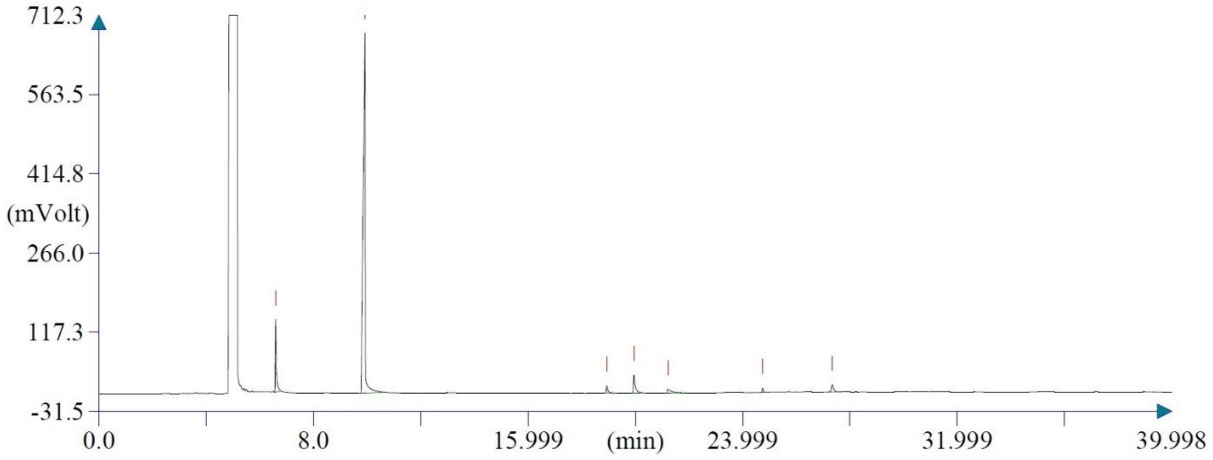

Reaction of phenylacetylene and 3-methylaniline

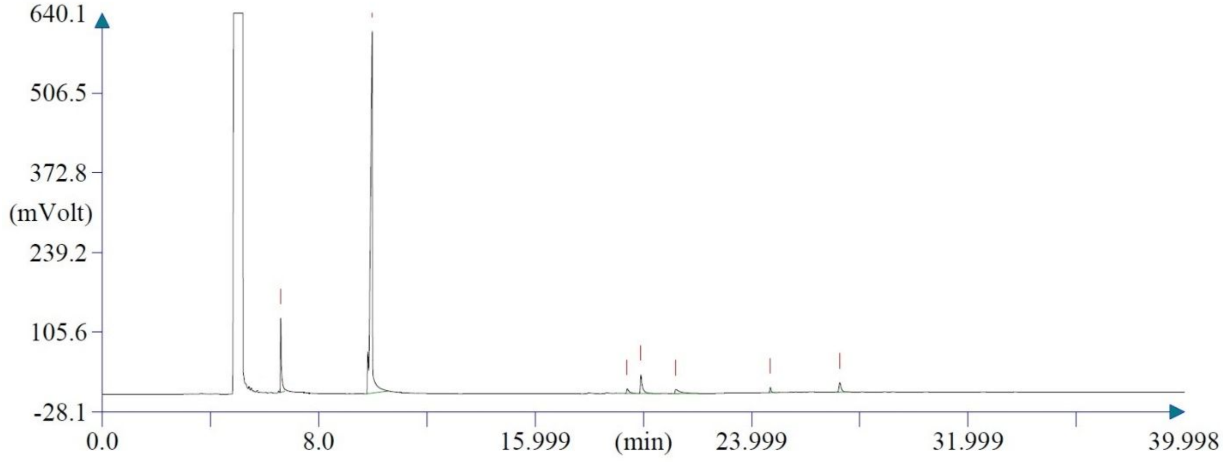

Supplement: Supplementary file 1 [file ja4c13481_si_001.pdf]
